# Supplementary figures and images for: Neural traces of composite tasks in complex task representation in the human brain reflects learning performance
Source: PLoS Biol. 2026 Jan 16;24(1):e3003613. doi: 10.1371/journal.pbio.3003613 (PMC12826513; doi:10.1371/journal.pbio.3003613)

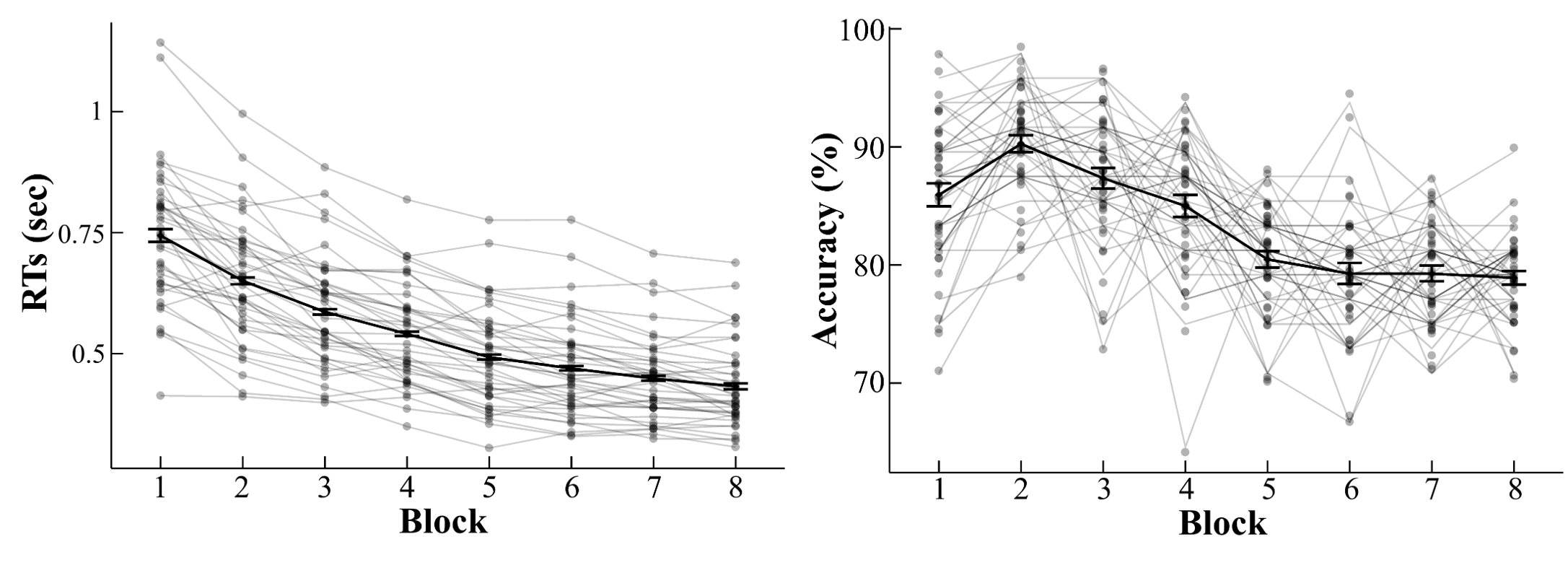

Supplement: S1 Fig — Individual RT (left) and accuracy (right), imposed with the group mean and SEM, are plotted as a function of block. Participants showed faster RTs over blocks on correct trials, while the accuracy declined after block 2. The decline in accuracy may be attributed to the decrement of the response deadline due to the staircase procedure of adjusting response deadline. Data underlying this figure can be found in the OSF repository (https://doi.org/10.17605/OSF.IO/MZF4A). (TIF) [file pbio.3003613.s001.tif]

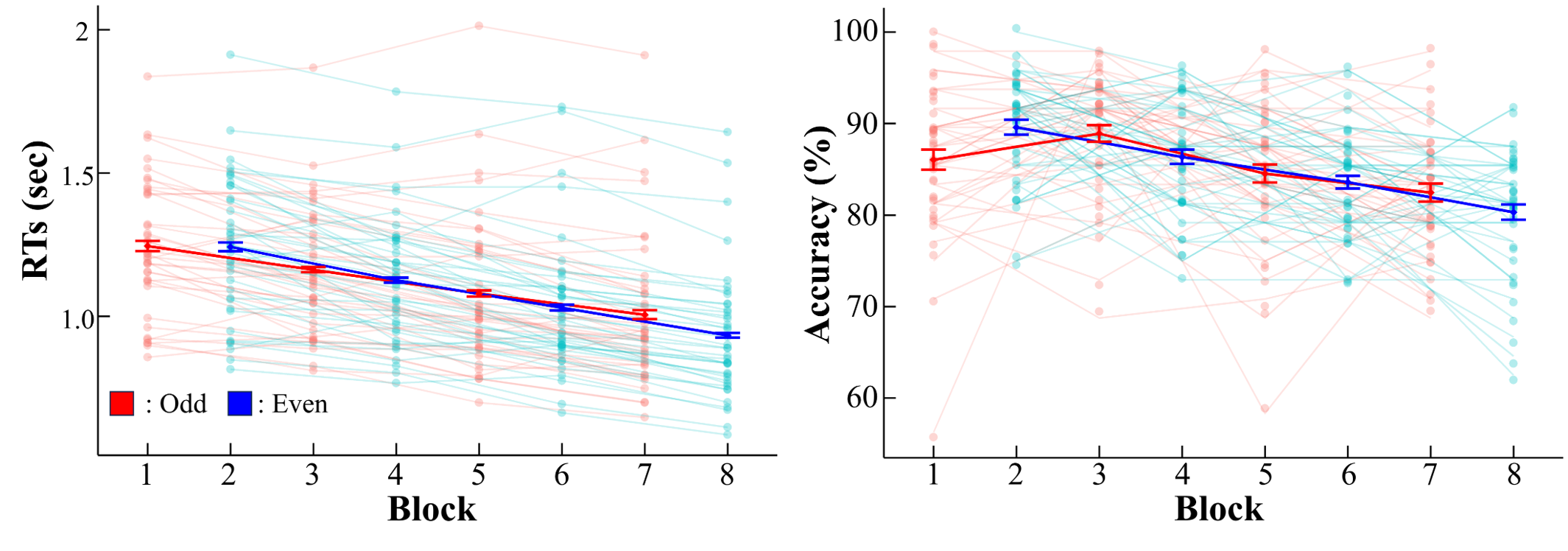

Supplement: S2 Fig — Individual RT (left) and accuracy (right), imposed with the group mean and SEM, are plotted as a function of block. Participants showed faster RTs over blocks on correct trials, while the accuracy declined after block 3. The decline in accuracy may be attributed to the earlier response deadline due to the staircase procedure of adjusting response deadline. Data underlying this figure can be found in the OSF repository (https://doi.org/10.17605/OSF.IO/MZF4A). (TIF) [file pbio.3003613.s002.tif]

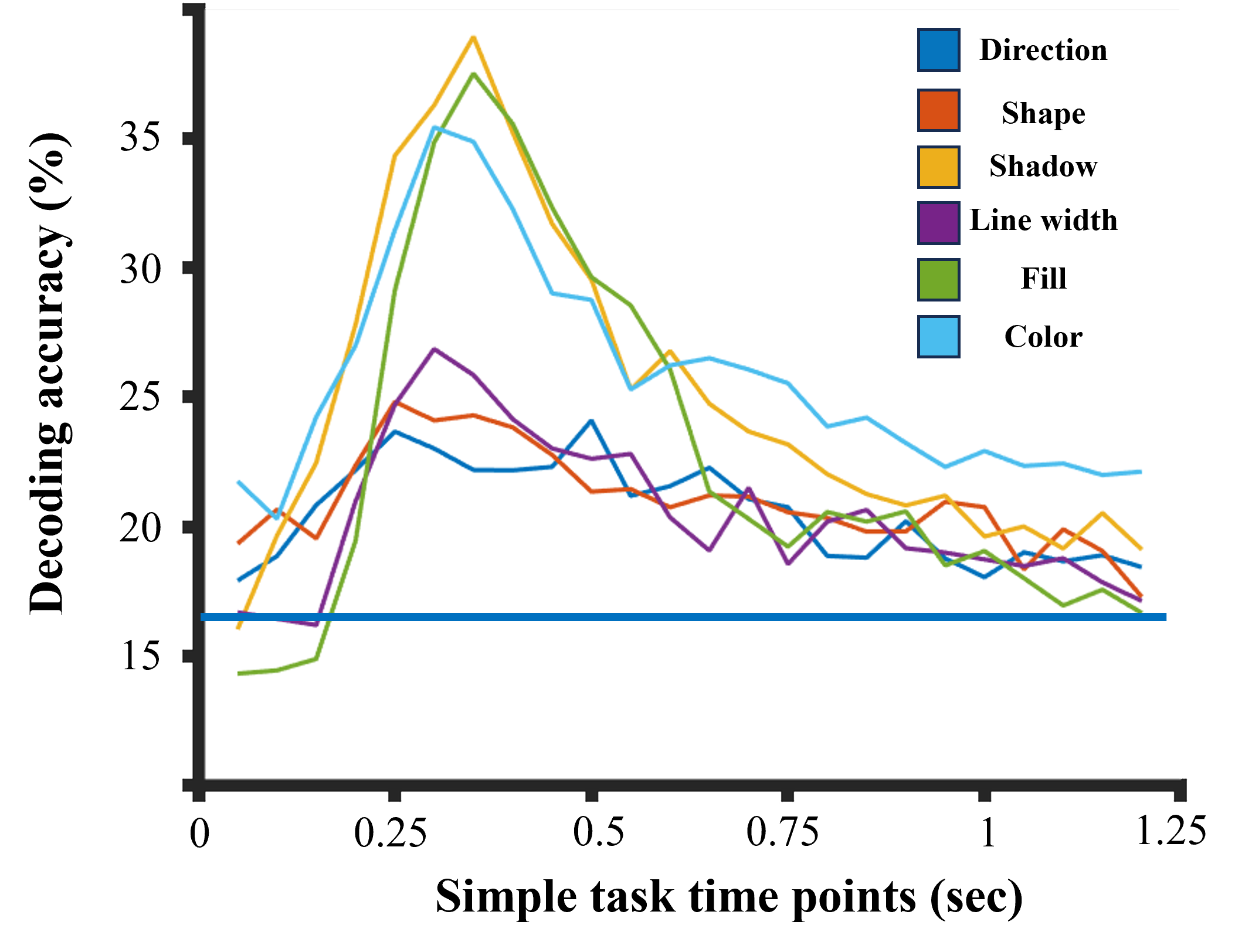

Supplement: S3 Fig — Blue flat line indicates chance-level decoding accuracy. Data underlying this figure can be found in the OSF repository (https://doi.org/10.17605/OSF.IO/MZF4A). (TIF) [file pbio.3003613.s003.tif]

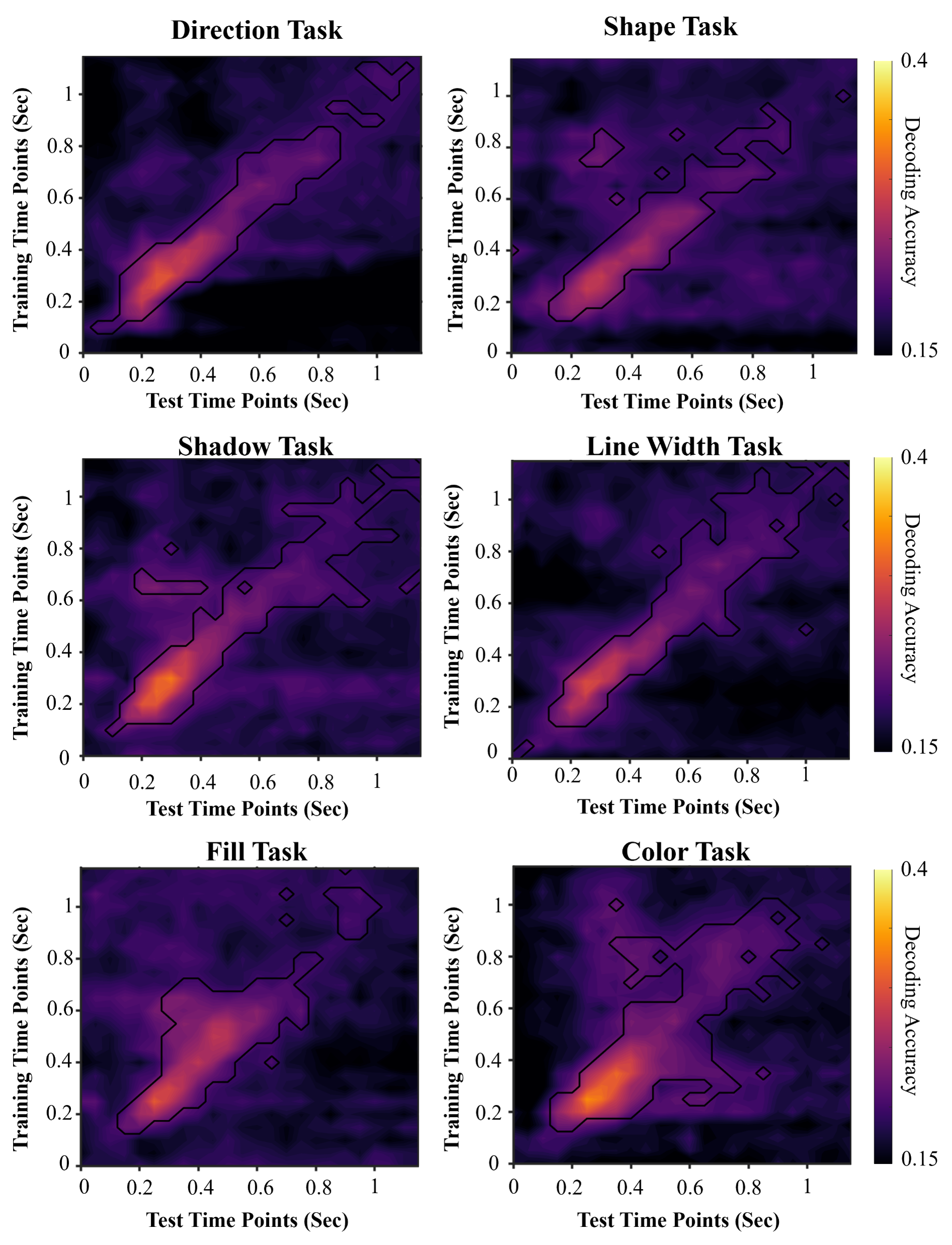

Supplement: S4 Fig — Black contour highlights clusters that showed significant time points after FDR correction. Data underlying this figure can be found in the OSF repository (https://doi.org/10.17605/OSF.IO/MZF4A). (TIF) [file pbio.3003613.s004.tif]

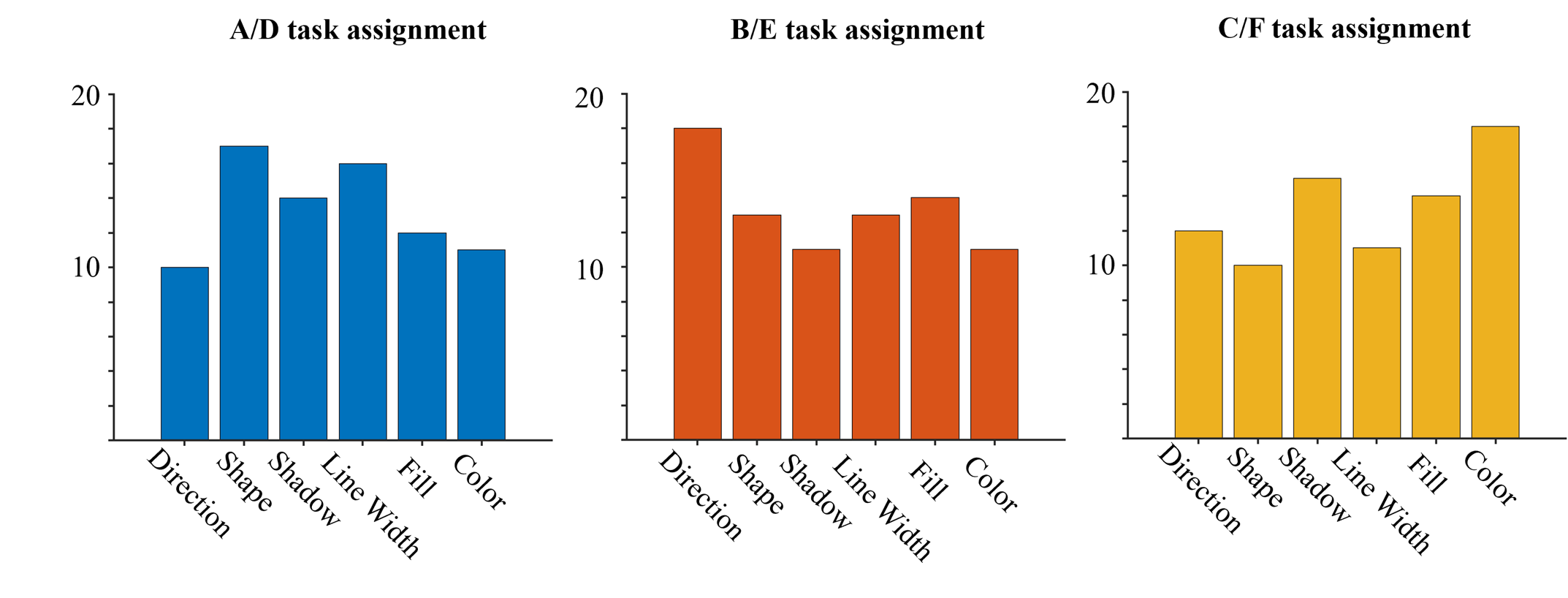

Supplement: S5 Fig — Data underlying this figure can be found in the OSF repository (https://doi.org/10.17605/OSF.IO/MZF4A). (TIF) [file pbio.3003613.s005.tif]

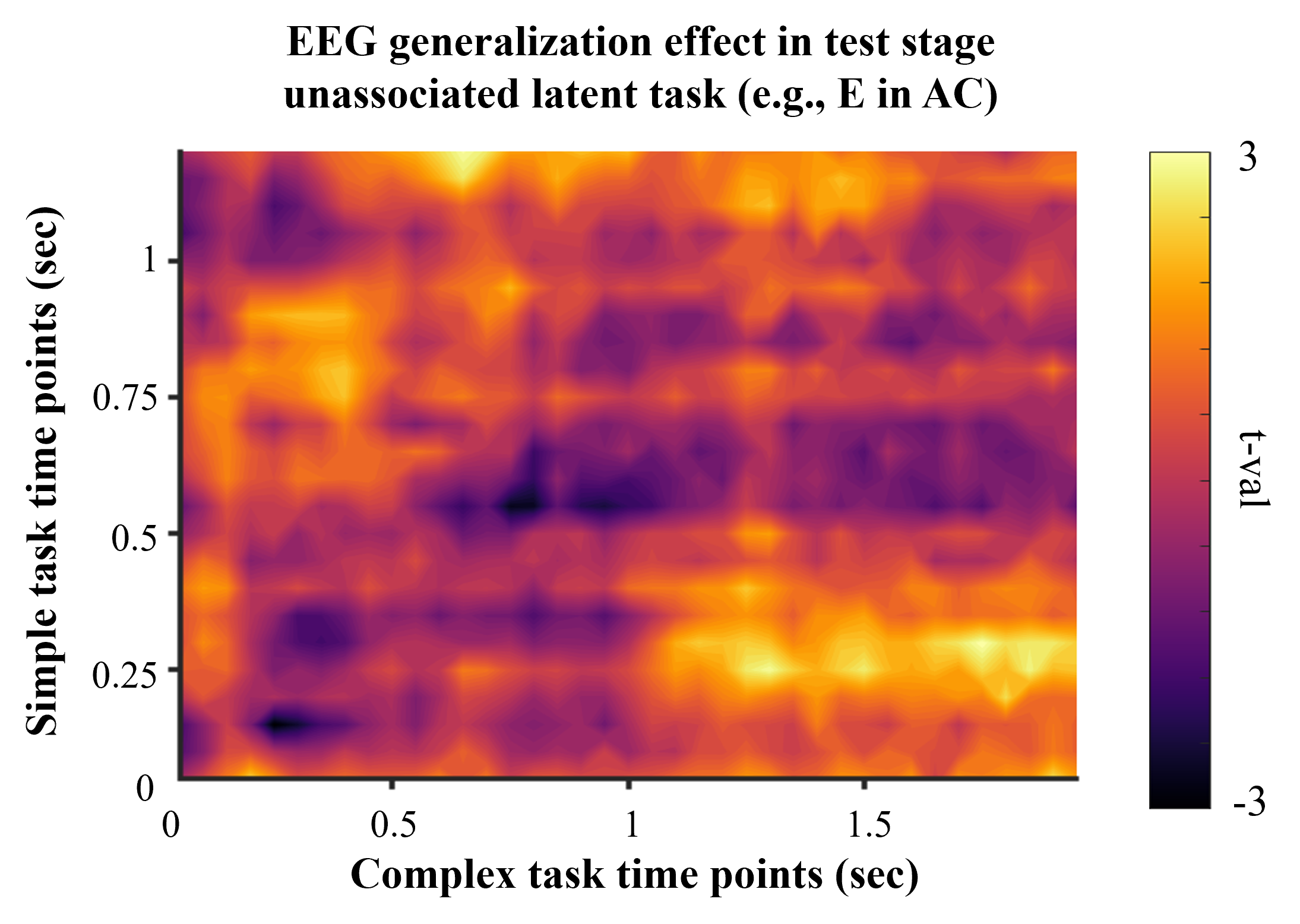

Supplement: S6 Fig — The result shows positive values between 1,000 and 2,000 ms in complex task EEG data, which partially overlaps with post-hoc EEG generalization analysis (Fig 2D). However, the largest cluster in the time window did not survive permutation test (p = .120). This result provides preliminary evidence that latent task reactivation after 1,000 ms in complex task EEG data might be confounded by the effect caused by higher presentation frequency of latent task (e.g., simple task B presented twice often compared to A or C during training stage). Data underlying this figure can be found in the OSF repository (https://doi.org/10.17605/OSF.IO/MZF4A). (TIF) [file pbio.3003613.s006.tif]
