# Supplementary material for: Neural traces of composite tasks in complex task representation in the human brain reflects learning performance
Source: PLoS Biol. 2026 Jan 16;24(1):e3003613. doi: 10.1371/journal.pbio.3003613 (PMC12826513; doi:10.1371/journal.pbio.3003613)
Supplement: S1 Table — Values are reported as the ‘mean (standard error of mean)’. Data underlying this figure can be found in the OSF repository (https://doi.org/10.17605/OSF.IO/MZF4A). (DOCX) [file pbio.3003613.s007.docx]

**S1 Table**
Behavioral performance of each simple task in the simple task phase.

|  | Tilt Direction | Shape | Shadow | Line Width | Fill | Color |
| --- | --- | --- | --- | --- | --- | --- |
| Acc (%) | 86.3 (0.87) | 90.6 (0.67) | 84.2 (0.95) | 80.6 (1.15) | 64.1 (1.20) | 94.1 (0.56) |
| RTs (ms) | 535.5 (18.29) | 520.5 (15.95) | 577.1 (15.47) | 574.7 (16.77) | 652.9 (17.24) | 478.9 (13.70) |

*Note**.* Values are reported as the ‘mean (standard error of mean)’. Data underlying this figure can be found in the OSF repository (https://doi.org/10.17605/OSF.IO/MZF4A).
