# Supplementary material for: Neural traces of composite tasks in complex task representation in the human brain reflects learning performance
Source: PLoS Biol. 2026 Jan 16;24(1):e3003613. doi: 10.1371/journal.pbio.3003613 (PMC12826513; doi:10.1371/journal.pbio.3003613)
Supplement: S2 Table — Values are reported as the ‘mean (standard deviation)’. Data underlying this figure can be found in the OSF repository (https://doi.org/10.17605/OSF.IO/MZF4A). (DOCX) [file pbio.3003613.s008.docx]

**S2 Table**
VIF scores of behavioral confounding regressors

|  | Response | Response Repetition | Post-error trials | Cue modality | Task repetition | Complex Task 1 (AC) | Complex Task 2 (DF) | Complex Task 3 (AD) | Complex Task 4 (CF) |
| --- | --- | --- | --- | --- | --- | --- | --- | --- | --- |
| VIF score | 1.09 (0.06) | 1.10 (0.08) | 1.08 (0.05) | 1.06 (0.03) | 1.05 (0.02) | 1.32 (0.14) | 1.31 (0.12) | 1.29 (0.11) | 1.32 (0.13) |

*Note.* Values are reported as the ‘mean (standard deviation)’. Data underlying this figure can be found in the OSF repository (https://doi.org/10.17605/OSF.IO/MZF4A).
