# Supplementary material for: Neural traces of composite tasks in complex task representation in the human brain reflects learning performance
Source: PLoS Biol. 2026 Jan 16;24(1):e3003613. doi: 10.1371/journal.pbio.3003613 (PMC12826513; doi:10.1371/journal.pbio.3003613)
Supplement: S3 Table — Values are reported as the ‘mean (standard error of mean)’. Data underlying this figure can be found in the OSF repository (https://doi.org/10.17605/OSF.IO/MZF4A). (DOCX) [file pbio.3003613.s009.docx]

**S3 Table**
Raw reaction times of test stage by block (in ms)

|  | Block 9 | Block 10 | Block 11 |
| --- | --- | --- | --- |
| Generalizable | 977.44(7.12) | 937.22 (6.31) | 909.07 (8.01) |
| Control | 985.58 (10.79) | 950.14 (7.12) | 922.89 (8.45) |

*Note.* Values are reported as the ‘mean (standard error of mean)’. Data underlying this figure can be found in the OSF repository (https://doi.org/10.17605/OSF.IO/MZF4A).
