# Supplementary material for: Neural traces of composite tasks in complex task representation in the human brain reflects learning performance
Source: PLoS Biol. 2026 Jan 16;24(1):e3003613. doi: 10.1371/journal.pbio.3003613 (PMC12826513; doi:10.1371/journal.pbio.3003613)
Supplement: Attachment — Submitted filename: Response_letter_final2.pdf [file pbio.3003613.s010.pdf]

Reviewer #1: Lee et al. focus on the question of how we learn to solve new tasks and suggest that this can be achieved using simpler tasks. Using a clever experimental design and EEG recordings, the authors show that during new complex tasks, simpler tasks are reactivated. However, what is most striking is that latent tasks associated with the simpler ones (which are not directly needed) are also reactivated, pointing to new insights about simpler tasks are actually reused for generalisation. The main finding is highly intriguing. There are a number of additional analyses and clarifications I would recommend to the authors to strengthen their work.

Response: We appreciate reviewer's positive evaluation of our manuscript and valuable feedback that helps improve this manuscript. Below we included point-by-point responses to the comments.

There are several additional analyses that would be important to establish the specificity of the generalisation effect:

1. Could non-associated simple tasks be decoded during complex tasks? For example, could task E be decoded during the AC task and could task B be decoding during the DF task?

Response: Thank you for your insightful comment. To address the concern, we measured the reinstatement of non-associated latent task (e.g., E in AC complex task) using the identical procedure to the associated latent task. Here, we found a positive unassociated latent task representation (e.g., above-chance decoding of E in AC) in 1,000 to 2,000 ms time points in complex task EEG data, which is similar to the time window of previously reported EEG generalization effect. However, the largest cluster in the time window did not reach significant level after permutation test ($p_{corrected}$ = .120), More importantly, this unassociated latent task reactivation was not evident between 850 – 1,000 ms in complex task EEG data, which showed stronger reinstatement of the associated latent task than the non-associated one (see response to Comment #2). The results are reported in the below and in the revised manuscript as figure 2C (right panel) and S6. Given that much of the time window showing this effect is after the response, when no task is actively being executed, we think the observed above-chance decoding reflects stronger off-task reactivation of the non-associated latent task, possibly due to the fact that the latent task (B or E) was presented twice as frequently as the other tasks during the training stage. To address the confounding factor of presentation frequency (e.g., B presented more often than A or C during training stage), we followed the reviewer's next comment and changed our analysis by using the non-associated latent task as a baseline.

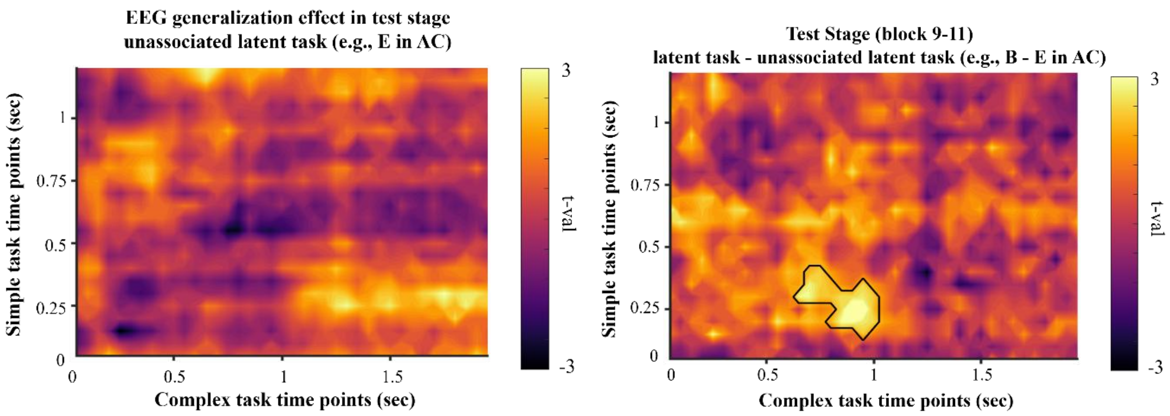

2. Once you have that information, you can then test whether the strength of the latent task (i.e. B during AC or E during DF) was significantly higher than the non-associated simple tasks (i.e. E during AC and B during DF)? The authors already show that latent tasks can be decoded above chance, but these analyses would test whether the expected latent task is activated more strongly than a non-associated simpler task. As far as I understand it, this would also be predicted by the theories the authors describe.

Response: Thank you for this important suggestion. Following your advice, we directly compared the decoding strength of the associated latent task (e.g., B during AC) against that of the non-associated task (e.g., E during AC) using paired t-test. A cluster-based permutation test revealed that the activation of the relevant latent task B was indeed significantly stronger than that of the non-associated task E during the 850-1,000ms ($p_{corrected}$ = .048). We believe this result reflects the EEG generalization effect specific to the associated latent task than non-related latent task, while the previous EEG generalization effect (only B in AC) might be confounded by the higher presentation frequency of the latent task during the training stage. Thus, we now use this contrast of associated vs. non-associated latent task as our main EEG test for the generalization effect. We also use this result for additional following analyses, including the correlational analysis. The following result, statistics, and detailed method are reported in the revised manuscript on p. 15 with following sentences:

Consistent with the prediction, latent task (e.g., B in AC) representations, compared to non-associated latent task (e.g., E in AC), were better decoded between 850ms to 1,000 ms post target onset in the test stage using decoders trained between 200 ms to 300 ms post target onset (Fig. 2C, right panel, $p_{corrected}$ = .048). While both theories predict that latent task represent should be presented in the test stage, integrative encoding theory further posits that integrative memory can form when participants experience both complex tasks that shares a simple task(11, 22); e.g., AB and BC) during the training stage. This suggests that generalization may occur during the training stage, starting from the third block. To test this prediction that generalization occurred during the training stage, we tested the decoding of the latent task

in the training stage (e.g., C from AB). In contrast to the test stage data, no time points reached statistical significance after the cluster-based non-parametric permutation test.

3. The authors examine how well could be the latent tasks (B and E) be decoded during the control tasks in Fig. S5. I would recommend explaining this in more detail in the main text and including the relevant statistical results.

Response: Thank you for this recommendation. We added a paragraph in discussion section for more detailed explanation. To summarize, we do not exclude the possibility of latent task reactivation in control complex tasks. Rather, we argue that there might be evidence of latent task reactivation in control complex task because the constituent tasks in a control complex task are also associated with (different) latent tasks. While we found numerically above-chance decoding of latent tasks in relatively late time points in complex task EEG data during control complex task (1,000 – 2,000ms), the largest cluster in the time window did not survive cluster-based permutation test ($p_{corrected}$ = .190), which prevents us from further interpretation. Furthermore, our additional analysis mentioned in response to the reviewer's comments #2 suggests that the EEG generalization effect in later time points in complex task EEG data might reflect potential confound of higher presentation frequency of latent tasks in the training stage. Supporting this hypothesis, the averaged latent task reactivation using the time window between 200 – 300 ms in simple task EEG data (for decoder training) and 850 – 1,000 ms in complex task EEG data (for decoder test, which showed significant EEG generalization effect in the new analysis; see responses to the reviewer's comment #1 and #2) did not show significant difference from zero ($t(39) = 1.63$, $p = .110$). These results are now included in our main manuscript, Fig. 2E and on p. 16:

As the control complex task also contained simple tasks with learned associations (e.g., A-B and D-E in AD complex task), it is possible that these latent tasks (B and E in AD) were reactivated by these associations. To test this, we regressed the logits of decoding performance for each task against a linear model with regressors for association and generalization effects in control complex tasks, and tested decoding performance of the latent simple tasks in test stage complex task EEG data (Fig. 2E). None of the cluster survived after controlling for multiple comparisons ($p_{corrected}$ = .190).

4. Following the point above, the authors state that decoding evidence for the latent tasks (B and E) was weaker during the control tasks (AD and CF) than the generalisable tasks (AC and DF). I might have missed it but I could not see formal statistical analysis supporting this claim. The authors should run and report a direct comparison testing whether decoding strength for the latent tasks is higher during the generalisable tasks, compared to the control tasks.

Response: We appreciate the suggestion for the confirmation analysis. We predicted that latent task reactivation would be weaker in control than generalizable complex tasks. This is because the constituent tasks in a control complex task are associated with different latent tasks, which may lead to interference during retrieval. On the other hand, both constituent tasks in a generalizable complex task are associated with the same latent task, which may provide a stronger cue for retrieval. Following the reviewer's comment, we formally tested this prediction that the latent task is more strongly reinstated in generalizable complex tasks than control complex tasks, by averaging the latent task activations in the time windows that showed significant cluster in the main EEG generalization effect. While simple t-test analysis showed that the latent task activation is significantly greater than zero in generalizable complex tasks (t(39) = 3.60, p < .001), we found a non-significant effect in the control complex tasks (t(39) = 1.64, p = .110). However, the paired t-test did not show significant difference between generalizable and control complex tasks (t(39) = 0.57), p = .571). These results are now included in our main manuscript, on p. 16:

Additionally, we analyzed latent task reactivation by averaging its activity within the time window that showed significant EEG generalization effect in our main analysis (Fig. 2C, right panel). A t-test performed on these values revealed no significant effect (t(39) = 1.63, p = .110). However, the difference between latent task reactivation in control complex task and generalizable complex task did not show significant effect (t(39) = 0.57, p = .571). The results did not support the reactivation of latent tasks in control complex tasks.

5. There are some additional analyses and clarifications important for understanding the connection between neural coding and behaviour:

One thing that is particularly interesting is that the latent task is not needed to solve the complex task and could in fact interfere with it, since it could direct attention towards irrelevant target features. Yet, stronger latent task coding was found to correlate with better learning performance (a faster reduction in RT in generalisable tasks compared to control tasks). The authors should elaborate on why stronger latent task coding aids (RT) performance, despite its potential for increased interference.

Response: We thank the reviewer for the insightful comment regarding the interpretation of correlation between EEG generalization effect and learning slope. We strongly agree with the reviewer's comment that the reinstatement of latent task representations during generalizable complex tasks could cause potential interference, as the latent task is not required to perform the current trial. This argument is also aligned with our behavioral experiments reported in our previous published paper with highly similar behavioral paradigm (Lee et al., 2022; Exp 2a: n =

50, Exp 2b: n = 84), which showed significantly higher intercept of RTs, possibly reflecting worse performance, for the generalizable tasks than control tasks in early trials of the test stage. This pattern also was also hinted in the current behavioral data, showing slightly higher residual RTs in the generalizable tasks than control tasks, specific at the first block of the test stage, even though the effect was not significant.

We propose that while there might be an early interference effect, latent task activation supports the learning of generalizable complex tasks over time in longer time scales. To support this argument, we conducted an additional analysis, separating EEG generalization effect and residual RTs of generalizable trials into each block (block 1, 2, and 3), and tested if the EEG generalization effect in each block can predict the performance of current and future blocks. Consistent with the reviewer's comment and our previous behavioral data, we found a significant positive correlation between latent task reactivation in block 1 and residual RTs in block 1 ($r(38) = 0.36$, $p = .023$) and block 2 ($r(38) = 0.35$, $p = .028$), suggesting that stronger reinstatement of latent task in early stage is related to worse interference effect. However, more importantly, we found significant negative correlation between latent task reactivation in block 1 and residual RTs in block 3 ($r(38) = -0.51$, $p < .001$), which suggests that the latent task reactivation in block 1 predicts faster residual RTs in block 3. This result suggests that indeed the latent task activation might increase the interference at the beginning of learning generalizable complex tasks, but in longer time scales, it supports the learning of generalizable complex task.

Regarding possible mechanisms of generalization in this paradigm, one possible explanation of this finding is that the consistent latent task reactivation supports the inference of indirect associations between simple tasks in generalizable complex tasks by bridging their connections. The reinstated latent task may contribute to generalization by becoming the cue for the next the round of cued retrieval through the big loop in the MTL, suggested by big-loop recurrence theory (Koster et al., 2018). The theory suggests that the process of memory retrieval in the MTL does not end after the reinstatement, but recurs into the MTL iteratively, which can work as an additional cue for the future cued retrieval. In our experiment, the reinstated latent task may contribute to generalization by becoming the cue for the future cued retrieval through the big loop in the MTL. In turn, the constituent simple tasks will be reinstated in the next retrieval, adding to their representation strength and subsequently benefiting the learning of the generalizable complex tasks, a process consistent with the recurrent interaction theory. Alternatively, the reinstated latent task may facilitate integrative encoding involving the two constituent tasks,

thus accelerate generalization by strengthening the association between constituent tasks, a process that supports the integrative encoding theory.

The relatively long time-scale for the behavioral generalization effect is intriguing. To this end, we offer two speculations. First, because task performance involves procedural memory, which typically relies on gradual learning, generalization effect in task learning may be accumulative and requires longer time than declarative memory. Second, it may take time to resolve the interference before generalization effect starts to facilitate performance.

We included this additional analysis and discussion in our main manuscript in result section in Fig 3C (as well as the section below), and on p. 19 with following sentences:

The results above suggest a link between the reactivation strength of the latent task in the brain and the behavioral generalization effect in task learning. However, it remains unclear why the reinstatement of latent task representation, which is not required for the current complex task, benefits performance of generalizable complex tasks. One might hypothesize that the reinstatement of the unneeded latent task would interfere with complex task performance. To investigate potential interference and generalization effects and their relation to latent task reactivation, we conducted an additional cross-subject correlation analysis, using mean residual RT in generalizable complex tasks and EEG generalization effect in each block and tested whether the EEG generalization effect in earlier blocks covaries with residual RTs of the current or later blocks. We hypothesize that latent task reactivation in one block will be correlated with slower or faster RTs in another block as a result of interference or generalization effect, respectively.

Only the EEG generalization effect in first block showed significant correlation with residual RTs in each block, but the direction of the correlation varied with block. Specifically, we found a significant positive correlation between EEG generalization effect in first block and residual RTs in first block ($r(38) = 0.36$, $p = .023$) and second block ($r(38) = 0.35$, $p = .028$), consistent with an interference account that stronger reactivation of the latent task is linked to slower responses (Fig. 3C, left and middle panel). However, EEG generalization effect in block 1 showed a significant negative correlation with residual RTs in block 3 ($r(38) = -0.51$, $p < .001$), which suggests that participants with stronger latent task reactivation in block 1 showed faster residual RTs in block 3 (Fig. 3C, right panel). EEG generalization effect in other blocks failed to predict residual RTs. Overall, these results suggest that latent task activation evoke interference in short time scale but benefits their behavioral by supporting generalization in later stage.

in later stage.

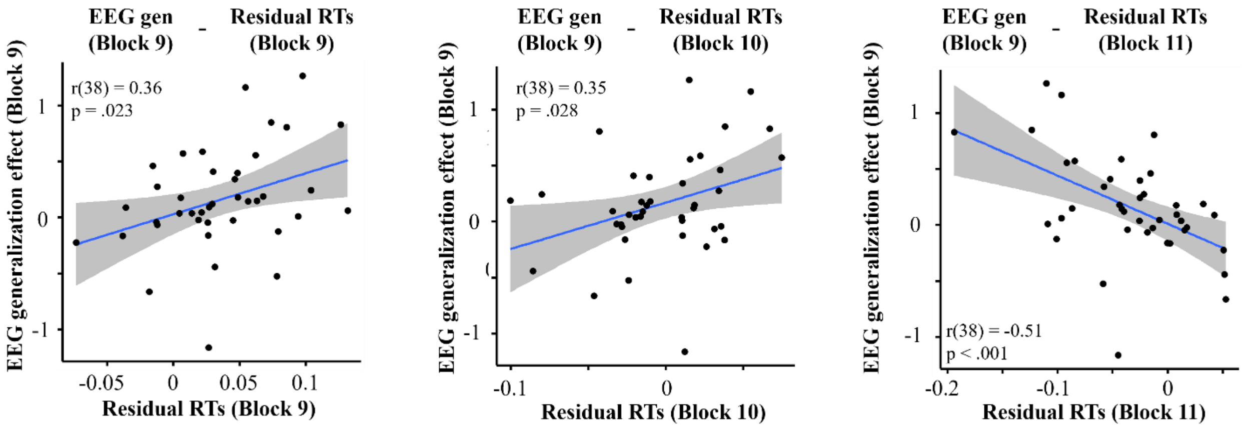

6. Performance analyses focused on RT there was a significant difference in this domain between the generalisable tasks and the control tasks. However, choice accuracy may be relevant to this process too. Does stronger activation of the latent task result in people being people are more likely to make errors (i.e. make an incorrect judgement about the target stimulus due to trying to enact the latent task)?

Response: Thank you for your insightful feedback. We conducted an additional analysis testing the correlation between participants' accuracy for generalizable complex tasks and EEG generalization effect in the time window of interest. We did not find a significant effect (r(38) = -.011, p = .506). This confirmation analysis suggests that EEG generalization effect is more strongly related to 'learning speed' itself, and it is unlikely that this finding is affected by a speed-accuracy trade-off. This result is added to our manuscript on p. 19.

The accuracy difference between generalizable and control complex tasks did not show any significant effect with EEG generalization effect (r(38) = 0.11, p = .506). This result indicates that reactivation of latent task in test stage supports the overall learning speed of generalizable complex tasks.

7. I might have missed it but I couldn't see a well-reasoned motivation or justification for the early vs. late separation procedure. Why was the 75% percentile of the RT distribution used? If the primary reason was because the generalisation effect spanned before and after the response, why not lock decoder testing to the response on each trial, and examine evidence before and after the response? Are the results the same as the current early vs. late separate method when you look directly at decoding evidence pre vs. post response?

Response: Thank you for your insightful comment. Our initial choice of the 75% RT percentile was a pragmatic decision, based on the assumption that a meaningful

generalization effect should precede the behavioral response. We considered this a reasonable cutoff to capture the majority of trials without being skewed by extreme RTs. During the revision process we also realized that, as pointed out by the reviewer and shown in our response to comment #1, the late reactivation effect may be driven by the higher presentation frequency of latent tasks in training stage. As a result, the later part of the previously reported generalization effect may not fully represent the reactivation of the latent tasks and hence may not be suitable for the correlational analysis. This may explain why in the original correlational analysis, the behavioral performance was only correlated with the EEG generalization effect from earlier time points.

Thanks to the reviewer's comment #2, we used the non-associated latent task as a baseline to test associated latent task reactivation. We note that this new analysis revealed an earlier time-window than the previous analysis. The temporal span of the new finding (850 - 1000ms) is close to the average RT (~950ms, c.f. Table S3), allowing us to drop the arbitrary choice of the cutoff and use the whole cluster for further analysis. This has not only resolved the issue the reviewer raised but has also significantly strengthened the core evidence supporting the behavioral relevance of the EEG generalization effect.

To strengthen the paper, more could be done to try and arbitrate between the two theories of associative memory:

8. The authors argue that both Integrative Encoding Theory and Recurrent Interaction Theory predict reactivation of the latent task during complex tasks and find some evidence that this is the case. The authors include one analysis that could in principle separate the theories: Integrative Encoding Theory predicts that latent tasks could be reactivated as early as the training stage. The authors do not find evidence for this prediction but the failure to detect this effect prohibits any strong conclusions. The fact that the authors find evidence for a prediction consistent with both theories opens the door to new experiments. However, I wonder if more can be done to discriminate the theories in the current data. Are there other tests that could distinguish the two theories the current data? If any can be devised they should be run, since this will greatly strength the paper.

Response: We thank the reviewer for this insightful suggestion to further explore our data. We strongly agree that adjudicating between the two theories in complex task learning is an important question. We also agree with the reviewer's comment that the integrative encoding theory would predict reinstatement of latent task in earlier stage, during training stage.

As the reviewer correctly commented, we could not find any significant cluster that survived cluster-based permutation test in the EEG generalization effect in training stage. Additionally, we conducted a more fine-grained analysis by separating the training stage into early (block 3-4), middle (blocks 5-6), and late (blocks 7-8) phase to better capture latent task reactivation more sensitively. However, we did not find statistically significant result in any phase, which overall, provides preliminary support for the recurrent interaction theory. Nevertheless, we acknowledge that a null finding is not conclusive to support one theory over the other. Additionally, the current study was designed to focus on the EEG generalization effect in the test stage rather than the training stage. We discussed this point in the revised manuscript on p. 21. This inconclusive finding also highlights the importance of reviewer's next question.

9. In addition to this point, I would recommend the authors include more critical discussion about how the two theories could discriminated in future experiments. What critical signatures would each have and what metrics could be used to distinguish them in future experiments on complex task learning?

Response: We appreciate the reviewer for the great suggestion. As we mentioned in the response to the reviewer's previous comment, we strongly believe that it is critical to distinguish between Integrative encoding theory and recurrent interaction theory in the task learning paradigm. To address the reviewer's comments, we added the following discussion to the revised manuscript on p. 22:

A key remaining question is how to distinguish between integrative encoding theory and recurrent interaction theory within our complex task learning paradigm. One possible way to dissociate two theories is by examining the timing of the reactivation of the latent task. The integrative encoding theory predicts that the latent task activation arises as early as the training stage, as the partially overlapped associations (e.g., A-B and B-C) are integrated into a conjunctive representation. On the other hand, the recurrent interaction theory posits that latent task reactivation occurs only at the test phase, when the generalizable complex task can be inferred. We did not find any significant clusters of latent task reactivation during the training stage, which is in line with the recurrent interaction theory. However, the null finding is not conclusive. Future studies designed to directly test this question are needed to address latent task reactivation in the training phase.

Another approach may be to examine the specific brain regions involved in generalization during task learning. A recent study(45) reported a functional gradient within the hippocampus, with posterior regions supporting smaller-scale associations (e.g., A-B, B-C) and the more anterior hippocampus region supporting more integrative representations (e.g., A-B-C) in narrative association task. Future neuroimaging studies with higher spatial resolution (e.g., those using fMRI) could provide more evidence that can distinguish two theories. Specifically, such studies could test whether the generalizable complex task learning relies on different subregions of hippocampus. For instance, involvement of the posterior hippocampal regions may suggest that generalization relies on local, small-scale representations, thus selectively supporting the recurrent interaction theory. On the other hand, engagement of the anterior regions may indicate that generalization is attributable to integrative representations, thus favoring the integrative encoding theory.

More minor points:

10. It would help to add absolute trial numbers (mean and SD) used in the decoding and RSA for clarity.

Response: We added the trial numbers (mean and standard deviations) we used in the decoding analysis and RSA in the main manuscript. Following information is now added to the revised manuscript in p. 13,

The mean (SD) number of trials included in the analysis was 293.48 (19.15) for the simple task phase, 215.05 (20.10) for the complex task training stage, and 52.68 (5.56) for the complex task test stage.

11. I might have missed it but were only correct trials were used for decoder training? The authors mentioned that the neural analyses controlled for RT. Did they also control for response accuracy? If not, the main results remain the same when this control is added?

Response: We only used the correct trials only. We clarified this point in our results and method sections in p. 13,

~~ we trained decoders on simple task phase EEG data, as described above, then tested on complex task phase data (see Method), only using correct trials in both simple tasks and complex tasks

12. Are the class probabilities required to sum to 1 with current decoding approach? This would be helpful to mention since it has implications for decoding strength of non-associated tasks. For example, if A, B and C are all above chance during complex task AC, there is very little probability left for the remaining classes and decoding evidence for those should therefore be low.

Response: The class probabilities indeed sum to 1 for each trial. However, we transformed these probabilities into logits (log-odds), which were used as the dependent variable in the RSA. As the logit transform is non-linear, the logits for all classes do not necessarily sum to 1. Following the reviewer's comment #2, the generalization effect now uses the non-associated task as baseline, which addresses the issue that the activation levels of constituent tasks would affect the baseline level. We directly mention this point in our methods and results section on p. 13:

For each test time point, the decoder produced a probabilistic prediction of each simple task being represented, which sums up to 1 across all simple tasks in each trial. We logit-transformed the decoding probability data, which prevent issue caused by the fact that sum of all classes' probability is 1.

13. In their introduction, the authors state that generalisation is based on association. I originally took this to mean 'similarity' and therefore felt this statement was overly broad,

since theories of generalisation tend to split into similarity based approaches and rule-based approaches. This is well described in Wu, C. M., Meder, B., & Schulz, E. (2024). Unifying principles of generalization: past, present, and future. Annual Review of Psychology, 76, a recent review of which I am not an author. I now see that the authors do not use association to mean similarity necessarily. Nonetheless, I would still recommend revising this sentence, since it sounds like you are saying all generalisation is based on association. That might not be true.

Response: We greatly appreciate the reviewer's insightful comment regarding the scope of the generalization in the current experiment. We strongly agree with the reviewer that not all generalization is based on association, and generalization can occur via different mechanisms.

We clarified the point that generalization can occur via multiple mechanisms, and that the current scope of the study is generalization that occur via associative inference on p. 3:

While generalization can occur via different mechanisms, such as rule-based or similarity-based (Wu, Meder & Schulz, 2024), generalization through association requires additional inferential processes as it allows for learning without direct association or experience.

14. Fig. 3A. 'Behavioral generalization effect'. It wasn't clear to me why this plot has the two bars of the same height, since the means and all data points sit within them and are different. If the two bars are not crucial, I would recommend removing them, so that more focus is on the data from the two conditions.

Response: We appreciate the reviewer for this detailed suggestion regarding Figure 3A. We agree that the two bars can cause misunderstanding about the time points used for the analysis. Their height was not meant to represent the data's magnitude, but rather to visualize the 'early' and 'late' time windows used in a previous version of our analysis.

When addressing the reviewer's comment (comment #1 & 2), we now use the latent task activation subtracted by un-associated latent task reactivation in the opposite sets (e.g., B -E in AC complex task) in all the correlation analysis. Following this change, we no longer separate the generalization effect into early and late periods. As a result, the bar graph has been removed from Figure 3.

15. The authors observed an unexpected increase in RT during the final test block for the control tasks. This unusual behaviour disappears when removing a particular control

regressor. Could this be due to multicollinearity? It could be useful to report correlations between regressors for the behavioural analysis (and RSA) and to report VIF scores in the supplement.

Response: Thank you for your insightful suggestion. After VIF analysis, we found that some of the confound regressors accounting for constituent simple tasks were highly correlated. For instance, in AC complex task, there were two regressors that accounting for each constituent simple tasks, A and C separately (to regress out potential between-task differences in simple task RTs), and the A regressor was shared by confound regressors in complex task AD trials. We performed new behavioral analysis by removing these confound regressors with high VIF scores (VIF > 2). This corrected analysis led to two important findings. First, our main behavioral finding is still robust, as we observe a significantly smaller slope, suggesting faster learning speed, for generalizable complex tasks than control tasks. Second, the unexpected increase in residual RTs in the final block for the control task is no longer present. Instead, we find gradual decrement of RTs over the blocks for both generalizable and control complex tasks. We now use this corrected behavioral result as our main finding and additional analyses. We reported average VIF scores and each confounding regressor in the supplemental materials as Table S2, and explicitly mentioned in the main manuscript in p. 10,

To control potential high multi-collinearity between confound regressors, we calculated variance inflation factor (VIF) scores for each regressor and confirmed that all VIF scores are lower than 2 (Table S2).

16. Related to the point above, were the main neural-behavioural correlations the same when using the regression slopes from the post hoc behavioural analysis where the control regressor was removed?

Response: In continuation with previous response, we conducted a new correlation analysis between new behavioral (after VIF correction) and EEG generalization effect (after subtracting latent task representation in opposite set [e.g., E from AC tasks]). Importantly, we still found significant negative correlation between behavioral and EEG generalization effect ($r(38) = -0.32$, $p = .043$), which suggests that the result of correlation analysis is not solely caused by the regressors with high VIF scores. This updated result is reported as the main analysis in our revised main manuscript and substituted for the previous correlation analysis.

17. Although many analyses use cross-decoding, some analyses train on EEG from a specific post-target window (e.g. 200-300ms). How were such training windows selected?

Response: The 200–300 ms window was selected based on the simple task decoding results: it was the window of peak decoding accuracy using simple tasks as both training and test data (with cross-validation, see Fig 2A). We additionally conducted a temporal generalization analysis (Fig. S4) using simple task EEG data, which also showed that the 200-300 ms time window is crucial for decoding the simple task EEG data. This window also corresponded to the time of the most robust latent task reactivation in our main cross-decoding analysis.

We have revised the result section to clarify that this was a pre-specified window for averaging, not a post-hoc selection for training with following sentences in page 18:

The decision to use 200-300 ms post-target onset for the simple task EEG data is further supported by simple task decoding analysis, which showed the most robust decoding performance in that time window (Fig. 2A and S4).

Reviewer #2: In this paper, Lee et al. investigate how the brain generalises simple task rules to more complex tasks. Participants first learn six simple tasks, then encounter complex tasks where pairs of those simple rules need to be combined. Importantly, they learn the complex task in an order that allows for inferring a link between two task sets (AB and BC). The authors then do cross-phase decoding where they train an EEG decoder during the simple task phase and test it during the complex task phase to show that the simple task B that links the complex task AC is reactivated during AC presentation.

The behavioural paradigm is elegant, the EEG decoding is technically impressive, and the results are interesting, particularly the reinstatement of latent task representations during novel task performance. Overall, this is a well-executed study.

However, in my view the interpretation that associative generalisation facilitates task learning is not strongly supported by the behavioural or neural data. Instead, what I think we see here is a spread of activity across associations, but not strategic generalisation. I elaborate on this point and other aspects that I think should be addressed in a revision in a bit more detail below.

We deeply appreciate your positive and constructive feedback. Below, we provide point-by-point responses to each of the comments.

Conceptual framing:
1. A core claim of the paper is that activating a latent task (B during AC trials) supports generalisation. But in this particular task design, activating latent B is not obviously useful to the participant. In classic associative inference paradigms, activating B helps you infer the link between A and C. This has instrumental value: it helps you solve a new problem (e.g., infer a relationship that helps you make better choices). In this task, there is no explicit benefit to recalling B during AC trials. In fact, recalling B might even be disadvantagoues due to increased interference of task-irrelevant information. Thus, the reinstatement of B in my view is much more likely to reflect automatic spreading of activation across associations rather than a functional inference process that helps participants perform the task better as it is implied in statements like this:"generalization can support the learning of a novel complex task if its associations can be inferred from other learned complex tasks."

Response: We appreciate the reviewer for the insightful comment and interpretation. We agree with the reviewer that the reinstatement of latent task is more likely to be an automatic process, rather than a strategic process that is consciously engaged to

improve performance. This automatic generalization via associative inference is indeed presented in previous memory literature (Zeithamova et al., 2012; Shohamy & Wagner, 2008; Liu et al., 2019), including the classic 'AB BC' paradigm, while the key difference is that the reinstatement of latent task is not directly related to the adaptive behavior in the current trial. Following the comment, we clarified in our discussion section that the latent task reactivation is likely to be automatic, rather than strategic, conscious inference process for efficient complex task learning in p. 23,

The interference effect (i.e., the significant positive correlation between EEG generalization effect in first block and the residual RT in the first and second blocks, Fig. 3C) observed early in the test phase suggests that the reinstatement of the latent task may not be strategic. Rather, it may reflect automatic cued memory retrieval, given that retrieving the associated item can be automatic after practice, such that inhibitory control is needed to suppress the retrieval(53, 54). In this study, the participants encountered each association (i.e., complex tasks) 96 times in the training stage. This level of exposure may facilitate automatic retrieval of the latent task in the test phase.

We added to the revised discussion on how automatic reinstatement of the latent task could benefit behavior, as detailed in the response to the next comment.

2. To make a convincing case for a generalisation mechanism of the sort implied by the authors, it would be helpful to run a trial-by-trial analysis time-locked to the response. Do participants respond faster on AC if B was reinstated more strongly compared to trials where B was not decodable? In the absence of such an effect or a similarly convincing analysis, I would find a framing in terms of a spread of activation across associations more appropriate than a framing in terms of generalisation.

Response: Thank you for this insightful comment. We strongly agree with the reviewer's comment that trial-by-trial analysis with the EEG data time-locked to the response would be helpful to strengthen the argument of generalization mechanism in our previous manuscript.

To directly test the reviewer's comment, we first conducted response-locked EEG analysis of the generalization effect. However, this analysis (see the figure below) did not reveal any cluster showing significantly better decoding of associated (e.g., B in AC complex task) than non-associated latent task (e.g., E in AC complex task). The null finding suggests that the observed latent task reactivation may not be related to the execution of the current trial. The null finding also prevents us from conducting the trial-level analysis suggested by the reviewer. As an alternative, we conducted a trial-by-trial analysis using latent task reactivation from stimulus-locked analysis in the time window of interest (Fig. 3C, right panel). We could not find any significant effect

suggesting that stronger activation leads to faster reaction times in a trial-by-trial level. However, the reviewer's comment and our own findings led us to an interesting new hypothesis: the generalization effect may support long-term learning rather than immediate, trial-by-trial performance.

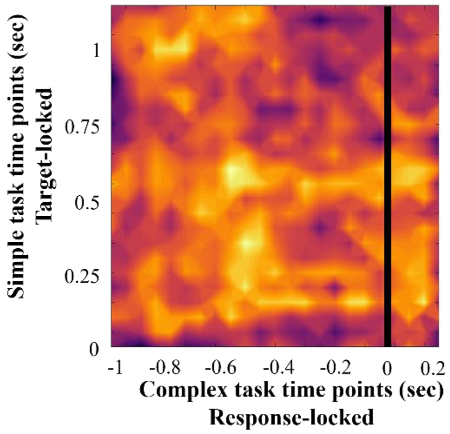

To test this hypothesis, we conducted an additional analysis, separating EEG generalization effect and residual RTs into each block (first, second, and third block) of the test stage, and tested whether the EEG generalization effect predicts the performance on generalizable trials of current or the future blocks using between-subject correlation. As expected, we found a significant positive correlation between EEG generalization effect of first block and residual RTs of first and second blocks (first block: $r(38) = 0.36$, $p = .023$, second block: $r(38) = 0.35$, $p = .028$), suggesting an interference effect that is consistent with the reviewer's comment #1 that the reinstatement of the latent task is not helpful with the performance on the current trial. However, we found significant negative correlation between EEG generalization effect of first block and residual RTs in the third block ($r(38) = =-0.51$, $p < .001$). This result is aligned with our behavioral data, showing numerically lower residual RT in generalizable than control complex task only in block 3, with the trend reversed in the first two blocks (Fig. 1E), although the difference is not statistically significant. The following results are included in the main manuscript in Fig 3c on p. 19.

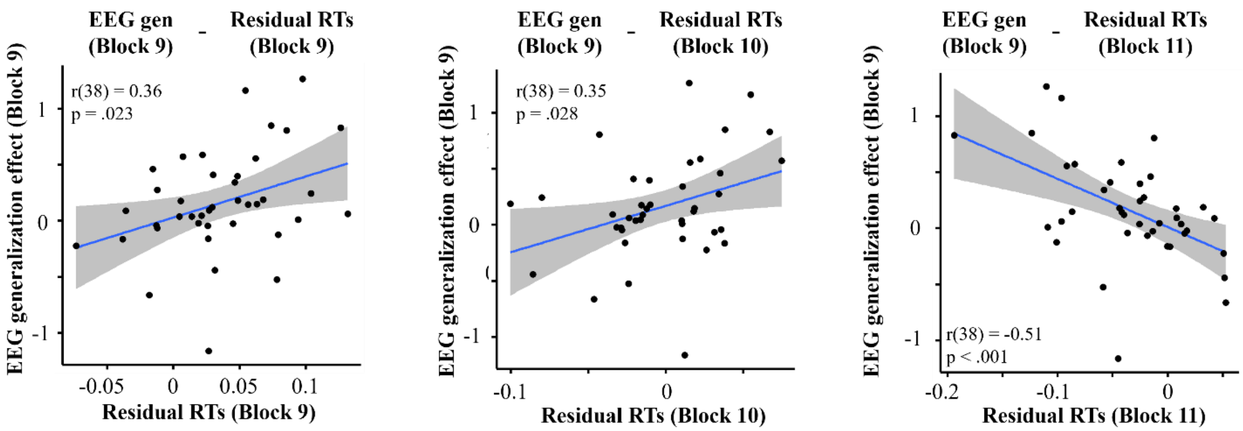

This result suggests that the latent task activation (i.e., the EEG generalization effect) in early trials does not benefit the performance on the current trial but rather supports the long-term learning of the generalizable complex tasks. In the revised manuscript, we speculate about the potential reasons for the slow generalization effect, as detailed in the response to the next comment. We also discuss potential mechanisms that convert automatic reactivation through associations into generalization in p. 22. The discussion reads:

The interference effect (i.e., the significant positive correlation between EEG generalization effect in first block and the residual RT in the first and second blocks, Fig. 3C) observed early in the test phase suggests that the reinstatement of the latent task may not be strategic. Rather, it may reflect automatic cued memory retrieval, given that retrieving the associated item can be automatic after practice, such that inhibitory control is needed to suppress the retrieval(53, 54). In this study, the participants encountered each association (i.e., complex tasks) 96 times in the training stage. This level of exposure may facilitate automatic retrieval of the latent task in the test phase.

The following analysis is included in the revised manuscript for further details in p. 18:

We expected that the EEG generalization effect would benefit performance of generalizable complex tasks, whether it is via stronger activation of integrated memory representation with pattern completion process (integrative encoding), or via stronger activation of conjunctive representation that is connected with latent memory representation throughout training(21). Therefore, we conducted exploratory analysis to investigate the behavioral relevance of the EEG generalization effect. We first assessed individual behavioral generalization effects using the RT slope difference between generalizable and control conditions (generalizable – control) and correlated this behavioral generalization effect with the EEG generalization effect from the previous analysis (Fig. 1E). The slope differences showed a significant negative correlation with the EEG generalization effect ($r(38) = -0.32$, $p = .043$), indicating that a stronger EEG generalization effect is linked to larger difference in RT decrease in generalizable tasks compared to control tasks. The accuracy difference between generalizable and control complex tasks did not show any significant effect with EEG generalization effect ($r(38) = 0.11$, $p = .506$). This result indicates that reactivation of latent task in test stage supports the overall learning speed of generalizable complex tasks.

Behaviour

3. The behavioural evidence the authors report is a faster RT slope in generalizable tasks compared to control tasks. There are a few problems I have with this. The first one is the underlying theoretical premise. Under standard associative memory or cognitive control models, I would expect interference when retrieving overlapping associations like AB → AC due to B intruding, especially early in learning. That should, if anything, lead to slower RTs for AC than for a novel pairing like AD. It is not clear to me in what way B helps bridge A and C in the context of this task.

Response: We appreciate the reviewer for the insightful comment regarding the interpretation of the correlation between the EEG generalization effect and the learning slope.

We strongly agree with the reviewer's comment that the reinstatement of latent task representations during generalizable complex tasks might cause a potential interference effect, as the latent task is not required for successful responses. This argument is also aligned with our behavioral experiments reported in our previous published paper with highly similar behavioral paradigm (Lee et al., 2022; Exp 2a: n = 50, Exp 2b: n = 84), which showed a significantly higher intercept of residual RTs for the generalizable tasks than control tasks in early test stage. This tendency was also found in the current behavioral data, where there were slightly higher RTs in the generalizable tasks than control tasks, specifically in the first block of the test stage (Fig. 1E), even though the effect was not significant.

This idea is also supported by our additional analysis in response to the reviewer's comment #2. To recap, in the block-wise correlation analysis between EEG generalization effect and residual RT in generalizable complex tasks, we found that stronger EEG generalization effect in block 1 is positively correlated with slower responses in generalizable complex tasks in both block 1 and 2, suggesting an interference effect as the reviewer predicted. However, the pattern was reversed in block 3, as a stronger EEG generalization effect in block 1 was linked to faster responses in generalizable tasks in block 3. Overall, the findings suggest that the reinstatement of the latent task may negatively impact performance on the current trial but benefit behavior in the long-term (e.g., after a few blocks).

We also added a section to discuss how latent task reactivation can lead to generalization (p. 21). Briefly, we offered two possibilities: (1) according to the big loop recurrence theory, the reactivated latent task can become the cue to retrieve the constituent tasks as additional teaching signals to associate the constituent tasks in

the generalizable complex tasks; and (2) the reinstatement of the latent task facilitates the integrative encoding of all three associated simple tasks.

4. Assuming this is a valid assumption - why is the RT improvement gradual over repeated exposure? If the participants indeed benefitted from integrating prior training, should this effect not be strongest immediately after the end of learning the AC associations?

Response: We agree with the reviewer's comment that the previous literature investigating the generalization found an immediate transfer of knowledge, or generalization when the design is focusing on specific memory items relating strictly to declarative memory, such as 'zero-shot' learning or 'one-shot' learning paradigm (Lake et al., 2015). To address this, we added the following discussion to the revised manuscript:

In contrast to the zero-shot learning findings in previous studies16-18 that showed immediate improvement in performance after successful inference, our analyses showed a gradual improvement of performance in generalizable complex tasks. We argue that this surprising finding could be due to two reasons. First, as we observed the reinstatement of the latent task is linked to both interference in early trials and generalization in late trials of the test phase (Fig. 3C), it is possible that the resolution of the interference hinders early manifestation of the generalization effect. Second, unlike previous studies, associative inference in this study applied to task performance, which involves procedural memory. As learning in procedural memory is typically gradual, we speculate that the reported generalization effect could be decelerated by the involvement of procedural memory. These points may result in slow generalization processes, contrasting to the fast generalization effects that are reported previously(16-18).

5. In general, the behavioural effects are not super convincing. The RT effect is statistically significant but quite modest in size, and the authors found no accuracy effects. It also seems to be driven by an increase in RT in the control condition in block 11 rather than a decrease in the generalisation condition. It would be very helpful to get a little bit more insight into the patterns of the behavioural data to assess potential floor effects, familiarity effects, or ceiling performance. At the moment this is not possible, because the behavioural data that is presented is minimal and highly processed. The authors should show raw RT and accuracy data, not just the residuals, across experimental phases (simple and complex tasks/train and test phases).

In general, are there any other confounds that could explain the RT benefit? Was task order (gen and con) counterbalanced across participants or could order effects / fatigue play a role?

Response: Thank you for your insightful and constructive comment. To address these concerns about potential confounds regarding the behavioral effect, we added the following clarifications to the revised manuscript.

Firstly, on the potential confounds of task order and fatigue, we clarify that the task order was fully randomized within each block, which means that generalizable and control complex task trials were presented equally frequently within each block of the test stage. This design was intended to minimize any potential order or fatigue effects that might systematically bias behavioral generalization effect.

Secondly, on the point that concerning the robustness of the behavioral effect: The reported faster learning speed in generalizable than control complex tasks echoes data from previous papers (Lee et al., 2022; Squire, 2004). In our own study (Lee et al, 2022), we reported the behavioral generalization effect in two independent experiments with same behavioral paradigm (Experiment 2A: n = 50, Experiment 2B: n = 84). Additionally, in both experiments the behavioral generalization effect was more pronounced in later than earlier part of the test stage.

Lastly, to address the concern regarding the main source of the RT effect and request for raw data, we re-examined our confounding regressors and removed those causing high multicollinearity (as indicated by VIF scores), which appeared to drive the RT increase in the control condition, to build a more robust model. After removing the regressor, we found that the RTs increment from block 2 to block 3 in control condition has been disappeared. The updated RT pattern is shown in Fig. 1E.

Even after removing those regressors with high VIF scores, we found significant learning speed effect and further found significant behavioral – EEG generalization effect correlation. We now use the corrected residual RTs for any further analysis and visualization. Additionally, we reported confounding regressors, their VIF scores, raw RTs, and accuracy data in Table S1, S2, ans S3.

6. A simple t-test on RT slopes ignores the repeated-measures structure. A mixed-effects model or repeated-measures ANOVA would be more appropriate, especially given the nested design (trials within blocks within participants).

Response: Thank you for this suggestion. To clarify our initial approach, the t-test was performed on the difference between the two learning slopes (generalizable vs. control) for each participant, making it conceptually equivalent to a paired t-test, or repeated-measures ANOVA with two conditions.

However, we fully agree that a linear mixed-effects model provides a more powerful and appropriate framework to directly account for the nested structure of our trial-by-trial data. Therefore, following the reviewer's recommendation, we conducted a confirmatory linear-mixed effect model analysis. We first created a linear mixed effect model using regressors in our main behavioral analysis (RTs ~ 1 + condition (gen vs con) * trial_index (RTs slope) + post_error + response_repetition + task_repetition + Cue_modality + (1 | Subject). In this model, the interaction term between condition and trial_index captures difference in learning speed (RTs slope) between conditions (gen vs con). We found a significant interaction term, which support our behavioral findings that RTs slope was significantly more negative in generalizable tasks than control tasks (t(4398.14) = -2.10, p = .036). An alternate model that includes slope as random effects still showed significant effect (t(4379.3488) = -2.055, p = .040), but the model failed to converge. For this reason, we decided to use the model without slope random effects. We included details on p. 10 of the manuscript:

To account for the nested design of the present experiment, we performed a linear mixed-effect model analysis (See Note S2) and found a consistent significant effect of learning speed (t(4398.14) = -2.10, p = .036).

Details are also included in the supplemental materials (Note S2) on p. 12.

EEG decoding

7. As far as I understand, the authors show decoding of B during presentation of AC. Do they correct for the reactivation of E on the same trials? It seems conceivable to me that both B and E are generally more active due to familiarity effects (they were both shown more often during training). It seems important to show that the reactivation is specific (e.g., B in AC and E in DF, but not B in DF, E in AC).
This is particularly true also for the reported correlation between the association effect (decoding constituent tasks) and the generalization effect (decoding latent tasks). These could in theory reflect more general factors such between-participant differences in SNR, attention or task engagement or other signal quality measures. Showing that the correlation is specific to the correlation between the association effect and the relevant latent task (B), and not there for the irrelevant latent task (E) would strengthen the overall conclusions.

Response: Thank you for your insightful and constructive comment. We strongly agree that the non-associated latent task (e.g., E in AC complex task) is a better baseline condition to assess the reinstatement of the associated latent task (e.g. B in AC complex task). To address the concern, we included the non-associated latent task as

a new regressor in the linear model for RSA and compared the regression coefficients between the associated and non-associated latent tasks using paired t-test. From this analysis, we found a significant latent task reactivation in test stage, located at 850 – 1,000 ms ($p_{corrected}$ = .048). Compared to previous reported results, the reactivation later in a trial (1000 – 2000 ms in complex task data) is no longer present. We argue that this change suggests that the previously reported late EEG generalization effect might be confounded by higher presentation frequency of latent tasks during the training stage (e.g., B presented twice often than A or C). For this reason, we use this new analysis as our main test for latent task reinstatement and used its result for all subsequent correlation analysis. All the figures have been updated in the revised manuscript as Fig 2C and S6, as you can see below.

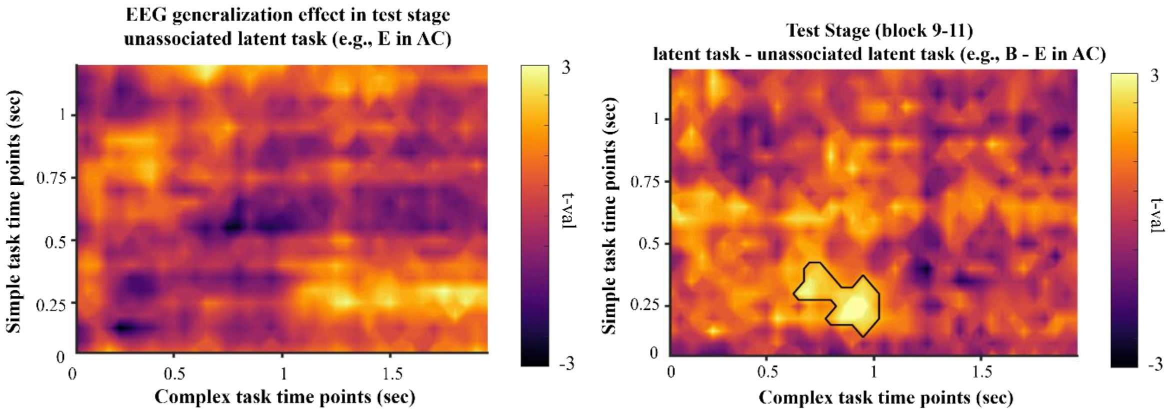

Additionally, the results have been updated in p. 15:

Consistent with the prediction, latent task (e.g., B in AC) representations, compared to non-associated latent task (e.g., E in AC), were better decoded between 850ms to 1,000 ms post target onset in the test stage using decoders trained between 200 ms to 300 ms post target onset (Fig. 2C, right panel, $p_{corrected}$ = .048). While both theories predict that latent task represent should be presented in the test stage, integrative encoding theory further posits that integrative memory can form when participants experience both complex tasks that shares a simple task(11, 22); e.g., AB and BC) during the training stage. This suggests that generalization may occur during the training stage, starting from the third block. To test this prediction that generalization occurred during the training stage, we tested the decoding of the latent task in the training stage (e.g., C from AB). In contrast to the test stage data, no time points reached statistical significance after the cluster-based non-parametric permutation test.

8. The authors use 1,100 ms post-target onset, the 75th percentile of all RTs across all participants, to split the EEG generalization effect into "early" and "late" windows. This fixed 1,100 ms split is problematic because it doesn't respect inter-subject variability in RT. Some participants may generally have much faster RTs than others, resulting in inbalanced assignment to early and late classes. This weakens the interpretability of the "early vs late" generalisation effect comparisons, because it means that different numbers of trials are assigned to each class for different individuals. To really argue that generalisation before

the response reflects latent reinstatement driving performance, the authors should analyse the time of decoding relative to the actual response, not just relative to fixed post-stimulus bins. A trial-by-trial analysis would be most convincing. Alternatively, the authors could use each participant's own 75th percentile RT to split their trials, so that "early" and "late" are relative to the subject-specific responses, not absolute.

Response: Thank you for your insightful comment.

Our initial choice of the 75% RT percentile was a pragmatic decision, based on the assumption that a meaningful generalization effect should precede the behavioral response. We considered this a reasonable cutoff to capture the majority of trials without being skewed by extreme RTs. However, we also strongly agree with the reviewer's opinion that the previous usage of overall 75% RT percentile is not capturing the individual RTs variabilities as RTs vary between participants

During the revision process we also realized that, as pointed out by the reviewer and shown in our response to comment #7, the late reactivation effect may be confounded by higher presentation frequency of latent tasks in training stage. As a result, the later part of the previously reported generalization effect may not fully represent the reactivation of the latent tasks and hence not be suitable for following correlational analysis. This may explain why in the original correlational analysis behavioral performance was only correlated with EEG generalization effect from earlier time points.

Thanks to the reviewer's comment #7 (using the non-associated latent task as a baseline to test associated latent task reactivation), we note that this new analysis revealed a time-window that is earlier than the previous analysis. The temporal span of the new finding (850 - 1000ms) is close to the average RT (~950ms, c.f. Table S3), allowing us to drop the arbitrary choice of the cutoff and use the whole cluster for further analysis.

By using a new time window that we found a significant cluster of EEG generalization, we still found a robust significant correlation with learning speed ($r(38) = -0.32$, $p = 0.43$) and EEG association effect ($r(38) = 0.36$, $p = .024$). Importantly, this time we included all time points (850 ms – 1,000 ms in complex task EEG data) in the correlation analysis. This finding adds another possible explanation why we found significant effect with early EEG generalization effect in our previous analysis: the early EEG generalization effect was less affected by the potential confounding factors mainly caused by the higher presentation frequency of latent task than the other simple tasks. Thanks to the reviewer's insightful comments, we have replaced our

previous analysis with a more robust and better-justified method. This has not only resolved the issue you raised but has also significantly strengthened the evidence presented in our manuscript. These findings and results are included in the results section.

9. Decoding accuracy and performance differ by simple task. Can the authors confirm whether simple task assignment to tasks A-F was properly counterbalanced? If more easily decoded features were overrepresented in B and E, decoding differences might reflect biases in decodability and not associative structure.

Response: We thank the reviewer for the insightful and detailed comments regarding the frequency of the simple task assignment to each label across participants. In our design, it is completely random which simple task is chosen as latent task or other association tasks. To confirm if the simple task assignment across participants were reasonably counterbalanced, we drew a histogram, presenting the number of participants assigned each simple task as 'A/D', 'B/E', and 'C/F' task. While the distribution is not perfectly uniform, we found that each simple task is reasonably well randomized, minimizing the concern that the main effects might be driven by a few simple tasks overly selected as a latent task. This figure is added below and included in the supplemental materials (Fig. S6) and cited in the main manuscript in p. 7:

We note that the assignment of each simple task to a specific label (e.g., A, B, C, D, E, or F) was always random for each participant. For additional confirmation, we visualized frequencies of each simple task assigned to each specific label (Fig. S6). This histogram showed no systematic bias of simple task assignments. This result supports that the reported findings are not mainly driven by a particular simple task.

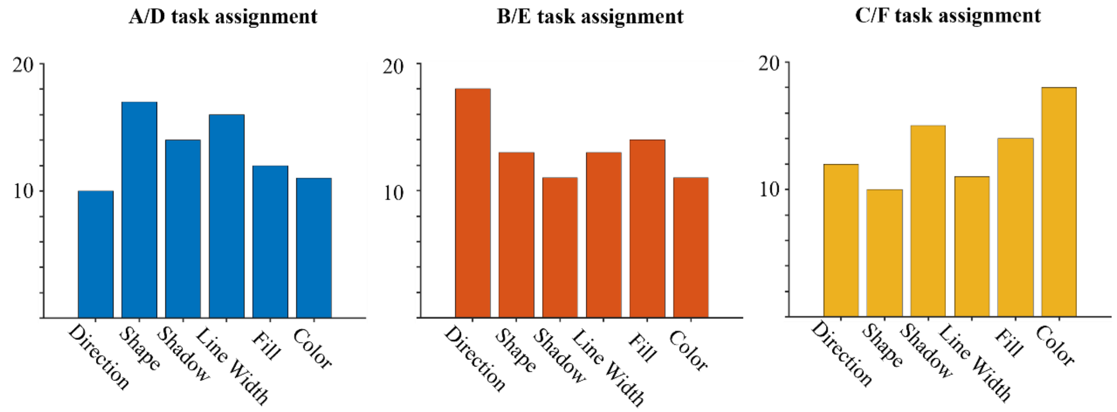

Minor points:

10. - What was chance performance in the complex task? How often was exactly one cue a match?

Response: Thank you for the clarifying questions. In this two-alternative forced-choice task, the chance performance was 50%. In more detail, the probability that exactly one of the two cues was a match was 50%. Similarly, the probability that both or neither of the cues were a match was also 50% (25% for both matching, 25% for neither matching). This point is now clarified in our revised manuscript.

11. - Was the order of Gen and Con blocks randomized or counterbalanced?

Response: Generalizable and control complex tasks were randomized within each block and presented equally frequently. We clarified in the revised manuscript that there was no generalizable blocks and control blocks that only presents generalizable, or control, complex tasks in the results section.

Reviewer #3: In this manuscript Lee, Hazeltine and Jiang describe an EEG experiment where participants completed a cued attention task with compound stimuli. In each trial, participants had to attend to two stimulus dimensions and make an XOR decision based on the features. Participants first trained on 'simple' tasks where they focused on just one stimulus dimension at a time. The main question of interest was whether participants reactivate representations of the component tasks (i.e. tasks or stimulus dimensions A, B and C) during these complex composite tasks (i.e. AB and BC) - and if they then reactivate 'latent' tasks that are associated through shared composite tasks (i.e. activate task B during an AC task). They find evidence that participants take advantage of the compositional structure of these tasks by learning to complete composite tasks that have a shared associate task faster than when these tasks do not sure an associate (i.e. faster learning on AC than AD).
They also demonstrate activation of these component tasks and of the latent tasks during completion of complex composite tasks. They demonstrate with evidence from individual differences that these processes are correlated across participants, and that activation of the latent task predicts the benefit of shared compositional task structure in behavior.

I thought that this was a very strong manuscript that uses a clever task to answer an interesting and important question. The results are quite convincing as well. I was a bit disappointed that the discussion did not do much to interpret these findings and their significance in linking concepts from associative memory theory to cognitive control. I also have some semantic gripes about the use of the term 'abstract' that I am not sure applies here. I also have some more minor points regarding the figures. However, overall, I thought this is a great study that makes an important contribution.

We thank the reviewer for the encouraging feedback. Detailed responses to each of the reviewers' comments are enclosed below.

1. The findings suggest that participants reactivate 'latent' tasks associated with the components of the current task- even though these aren't relevant to the task at hand. This is a pretty profound result - especially as there is no obvious direct benefit for reactivating information about the 'B' task on 'AC' trials. If true, I would think this means that we might expect that participants would show efficiency improvements in completing task B after completing an AC trial, for instance. It also means, more generally, that maybe the same medial temporal-mPFC network that is thought to be involved in this kind of associative learning might also be involved in completing this kind of inference and construction of new task representations. I am not sure if the authors agree with these ideas, and I don't think these are givens for much of the cognitive control community. I would like to see the

authors devote more space to fleshing out the significance of their findings to understanding the compositional nature of tasks and the implications of extending some of what we know about associative inference in the memory literature to the domain of cognitive control.

Response: We appreciate this insightful comment regarding implication of the study in terms of bridging associative inference in memory literature and cognitive control.

First, we agree with the reviewer's prediction that completing 'AC' trials might lead to subsequent performance benefits on task 'B' as a result of latent task reinstatement. First, the associative inference literature documents that the recall of item B improves after successful inference of indirect association between A and C. Secondly, in the classic task switching paradigm, it has been consistently reported that responses are faster when the task repeated, compared to when the task switched. If we interpret this result as recent reactivation of task representation (e.g., task inertia) facilitates the performance of the same task in the next trial, same logic should be applied in our study, such that if simple task B is reactivated, its reinstatement is expected to facilitate the performance of the same task on the next trial. This would be an interesting future direction to test whether reactivation of a task, rather than direct execution of it, benefits future task performance. As the reviewer pointed out, this could be a way to bridge memory and cognitive control research.

Secondly, we agree with the reviewer that MTL-mPFC network would be involved in the generalization process via associative inference in complex task learning. One influential theory (Vaidya & Badre, 2022) suggests that the MTL-mPFC network plays critical role in representing abstract task structures. This paper argues that the role of the MTL-mPFC network extends beyond its function in declarative memory. While traditionally known for building cognitive maps for spatial navigation, the authors further extended this view that this network also represents abstract task structures and concepts, thereby suggesting that this network also plays crucial role in procedural memory, which is strongly involved in task learning paradigm. By representing and organizing this abstract task structures into cognitive maps, it can further support the inference of new relationships between these task representations, for example, by using associative inference, or acquired equivalence. While the current study has its limitations in spatial resolution as we used EEG recording, we believe that MTL and mPFC network should involve in complex task learning, when associating simple task representation and forming cognitive maps of simple task representations. This cognitive map further supports the generalization

when learning generalizable complex task, by supporting associative inference using cognitive map of simple task representations.

Lastly, our study has its implications in bridging the literature on associative memory with cognitive control. Previous literature has provided convincing evidence for a division of labor: the MTL-mPFC network is involved in building a cognitive map of abstract task information, while the frontoparietal network implements this knowledge as actionable production rules to exert cognitive control. Our key finding of latent task reactivation during generalization bridges associative memory and cognitive control functions for adaptive behavior. This suggests that the cognitive map or networks of task associations, potentially stored in the MTL-mPFC, is dynamically accessed and translated into adaptive behavior via cognitive control function, potentially through the frontoparietal network. The following points are included in the discussion section in the main manuscript in p. 24:

The current study bridges a link between memory literature and cognitive control domain. The activation of associated memory items, even when not relevant to the current task, has been extensively reported in the memory literature(8, 39, 43) . The finding of activation of latent task activation shows that task learning may leverage the same cognitive and neural mechanisms that construct and navigate cognitive maps of abstract spaces to guide behavior. From this viewpoint, task representation can be modeled as a trajectory through a structured, map-like task space, mainly in hippocampal-orbitofrontal cortex network(23, 32, 56-58). The cognitive control function has been known to play critical role in translating information from cognitive maps into actionable production rules via the frontoparietal network(23, 32). The current study extends this finding by showing that cognitive control functions, which heavily involves lateral prefrontal areas, is supported by latent task reactivation through associative memory, which is believed to be supported by the medial temporal lobe (MTL).

2. I would also appreciate it if the authors could elaborate more on the cognitive processes taking place that give rise to the generalization effect. Since task B is not relevant to the performance of the AC task, what is it about evoking this 'latent task' that facilitates performance? Have participants formed some kind of multitask control policy in the AB and BC tasks that is being pulled back up here? Or is it something else?

Response: We appreciate the reviewer for insightful question regarding the cognitive process by which latent task reactivation supports faster learning of generalizable complex tasks.

For the reviewer's first point regarding the 'mechanism' of how latent task reactivation supports generalization, there are two possible mechanisms supporting our findings that latent task reactivation facilitates learning. One is the big-loop theory, feedback network that involves along the MTL (Koster et al., 2018). In this viewpoint, the reinstated latent task may send back to hippocampus through

feedback loop and then becomes the cue for the next round of cued retrieval, thereby facilitating the building of a new link between indirectly associated tasks. In this way, the reinstated latent task benefits the learning of the generalizable complex tasks. Alternatively, the reinstated latent task may facilitate integrative encoding involving the two constituent tasks, thus accelerating generalization by strengthening the association between constituent tasks. While dissociating these two possible theories in complex task learning should be the topic of additional studies, both theories support that the network between simple tasks in cognitive map lays foundation for efficient learning, and latent task reactivation facilitates new associations between indirectly linked tasks, via 'big-loop' mechanism or integrative encoding mechanism.

For the reviewer's second point regarding the 'multitask control policy', we argue that the training of AB and BC complex tasks might form procedural memories that include how to perform the two constituent simple tasks. This idea is supported by the behavioral result showing the gradual decrement of RTs over trials after the initial inference of indirect associations. Additionally, we found that the latent task reactivation in early blocks only supported performance of generalizable complex task in later blocks (Fig. 3C), which suggests that the behavioral benefit of latent task reactivation is gradual, rather than immediate. These results are somewhat inconsistent with the previous literature presenting immediate behavioral improvement after successful associative inference (i.e. zero-shot learning; Lake, Salakhutdinovm, and Tenenbaum, 2015; Lake, Ullman, Tenenbaum, 2017). This may be due to the fact that associative inference in this study applied to task performance, which involves procedural memory. As learning in procedural memory is typically gradual, we speculate that what participants formed during the training stage of complex task might be the updated procedural memory that could benefit their behavior in gradual manner, which might be linked to 'multitask control policy' as the reviewer suggested.

Another possible account to explain the gradual improvement of behavior after associative inference is that reactivation of the latent task supports the inference of compositional structure of task representation. Indeed, previous literature suggested that the compositional structure of task representation may has its benefits in flexibility and generalization, but it may cause an interference effect as one component is shared between different tasks (Badre, 2025). Our study further demonstrates the temporal dynamics of interference and generalization by showing that latent task reactivation leads to interference effect in early stage but also leads to gradual improvement in complex task learning in later stages. This gradual resolution

of interference effects driven by the compositional structure of memory components offers another pathway for bridging the fields of memory and cognitive control.

We included the following points in the discussion section in p. 23:

Because the reinstatement of the latent task is not required for the current complex task, the reactivation may cause interference in generalizable complex task performance. Indeed, previous studies have suggested that the compositional structure of task representation may benefit flexibility and generalization but may cause interference as one component is shared between different tasks(2, 32). Our study characterizes the temporal dynamics of interference and generalization by showing that latent task reactivation leads to interference effect in early stage but also leads to gradual improvement in complex task learning in later stages (Fig. 3C).

The interference effect (i.e., the significant positive correlation between EEG generalization effect in first block and the residual RT in the first and second blocks, Fig. 3C) observed early in the test phase suggests that the reinstatement of the latent task may not be strategic. Rather, it may reflect automatic cued memory retrieval, given that retrieving the associated item can be automatic after practice, such that inhibitory control is needed to suppress the retrieval(53, 54). In this study, the participants encountered each association (i.e., complex tasks) 96 times in the training stage. This level of exposure may facilitate automatic retrieval of the latent task in the test phase.

In contrast to the zero-shot learning findings in previous studies(16-18) showing immediate improvement in performance after successful inference, our analyses showed a gradual improvement of performance in generalizable complex tasks. We propose that this surprising finding could be due to two reasons. First, as we observed the reinstatement of the latent task is linked to both interference in early trials and generalization in late trials of the test phase (Fig. 3C), it is possible that the resolution of the interference hinders early manifestation of the generalization effect. Second, unlike previous studies, associative inference in this study applied to task performance, which involves procedural memory(55). As learning in procedural memory is typically gradual(6, 55), we speculate that the reported generalization effect could be decelerated by the involvement of procedural memory. These processes involving resolution of interference and learning of procedural memory may result in slow generalization processes, contrasting to the fast generalization effects that are reported previously(16-18).

3. Related to the above point, one potential limitation of this experiment is that the simple 'tasks' are essentially confounded with the different stimulus dimensions participants are asked to attend to. These 'tasks' could also be described in other terms like 'attentional sets.' Maybe the authors could acknowledge or debate this point in the discussion?

Response: We thank the reviewers for raising an important issue regarding the term 'task', and its relation to attentional set. We agree with the reviewer's point that the simple task is strongly intertwined with the stimulus feature participants need to focus on for successful response.

We would like to first clarify that the way we defined tasks is similar to the definition of tasks used by many other influential research on task switching (e.g., Rogers and

Monsell, 1995; Rubin and Meiran, 2005; Rubinstein, Meyer, and Evans, 2001). Additionally, given the available evidence regarding mixing costs and the effects of valency of the stimuli, it appears that the switch costs observed in tasks defined on relevant stimulus features do not merely reflect attentional processes, although they likely play a role.

In the revised manuscript, we explicitly defined the term task and discussed its relation with similar terms on p. 5:

While the 'task' in this paradigm is strongly intertwined with feature-based attention, our use of the term is consistent with classic task-switching literature(28-30), where a 'task' is requires the process that enables the efficient guidance of attention toward task-relevant feature. Furthermore, the task-switching literature provides convincing evidence that a 'task' cannot be fully explained by feature-based attention(29-32). For instance, mixing costs, which refer to the cognitive cost observed when performing a task in a mixed-task block (i.e., on each trial one of multiple possible tasks is performed) compared to a single-task block, cannot be fully explained by shift of feature-based attention, since the cost occurs even when participants repeat the same task, implying no change in feature-based attention (29, 30).

4. In the discussion (p. 20), the authors write: "In addition, the decoder was trained with simple task EEG data to exclude potential confounding effects caused by the response paradigm ('exclusive or' design) from the complex tasks, which suggests that the decoded information is abstract task information."

I agree that this procedure helps remove some of these confounds - but I am not sure it necessarily means that the decoded information is an "abstract task representation." The simple tasks themselves just require very concrete decisions about the stimulus features. The complex tasks are more abstract in that they require a second level of processing on the results of the simple tasks - but it seems likely that the decoded representations of the simple tasks here could just reflect this first stage of processing.

Response: We sincerely thank the reviewer for this insightful question. We strongly agree with the reviewer's point that the decoders, trained on the simple tasks, will reflect the early, concrete processes the relevant stimulus feature. However, we would like to clarify that the use of "abstract" is to emphasize the task representation that is not specifically tied to simple response mapping, or specific feature value (e.g., red color).

We clarified this definition of the term 'abstract' on p. 25:

One might interpret this decoded information as simply reflecting the concrete, early-stage processing of concrete stimulus features in simple tasks (e.g., the oval shape), rather than abstract type of representation (e.g., shape processing). While the decoded information likely involves early-stage

processing, we argue that this representation is abstract for two main reasons. First, the trained and tested EEG data do not share same response rules (simple task: ‘matching’ decision, complex task: ‘exclusive or’ decision), indicating that the decoded information is not specifically involved to a response related process. Second, the decoders were trained at the task level using trials with different stimuli. Thus, the decoded representation was not specific to a specific cue or feature value (e.g., ‘red’) but rather reflected the cognitive process of guiding feature-based attention along the encompassing feature (e.g., color). Overall, we argue that this representation is ‘abstract’ because it represents a higher-order rule that encompasses specific feature values and cognitive processes that flexibly guide these feature-based attention.

5. The discussion includes a reference to using recurrent neural networks and state space models to analyze these data on p. 21 but does not detail what these models are or how they are useful for answering these questions. Could the authors please either elaborate on this point or remove it?

Response: We thank the reviewer for this excellent suggestion to elaborate on the potential of modeling approaches for future studies. We have revised the discussion to provide more detail, as we agree this strengthens the paper.

The primary reason we suggest using recurrent neural networks (RNNs) or state-space models is that they offer a way to move beyond associative accounts and directly test for the compositionality of task representations.

Our current decoding and RSA analyses are powerful for demonstrating that task representations are separable and exist within a structured representational space. However, these methods provide relatively static, indirect measures of the underlying neural code. For instance, they show that a representation exists at a certain time, but not how it is dynamically transformed or combined with other representations.

In contrast, state-space models and trained RNNs allow us to capture the latent dynamics of neural activity. By analyzing the trajectories of neural population activity within this state space, we can ask more direct questions about compositionality. For example, one could explicitly test whether the neural representation for a complex task is a linear summation (i.e., vector addition) of the representations for its constituent simple tasks and processes (e.g., "Color" + “Pattern” + "Left button"). Finding such an algebraic relationship in the neural state space would provide direct evidence for compositional computation, a claim that is difficult to substantiate with decoding analysis or RSA alone.

While we found that the future direction of investigating compositionality using latent state-space modeling is promising, we decided to remove the part while

keeping the connection between the current study and compositionality of task representation. The following part is incorporated into the discussion section on p. 23,

Because the reinstatement of the latent task is not required for the current complex task, the reactivation may cause interference in generalizable complex task performance. Indeed, previous studies have suggested that the compositional structure of task representation may benefit flexibility and generalization but may cause interference as one component is shared between different tasks(2, 32). Our study characterizes the temporal dynamics of interference and generalization by showing that latent task reactivation leads to interference effect in early stage but also leads to gradual improvement in complex task learning in later stages (Fig. 3C).

6. I wasn't sure what the individual data points in Figure 3C and 3E represent. I am guessing these are time points? The current format is a little confusing because the adjacent panels show participants as individual data points. If these are time points, I would suggest connecting these points with a line, as there is a continuity between them (unlike individual subjects).

Response: We thank the reviewer for pointing out the lack of clarity in Figures 3C and 3E. The reviewer is correct; each data point in these panels represents a time point, and we agree that the original format was potentially confusing in contrast to the adjacent panels.

The figure has been removed due to the new analysis we adopted. Specifically, one potential confounding factor in EEG generalization effect reported in the previous version is that the latent tasks (B and E) were presented twice as often as other simple tasks during the training stage. To control for this frequency effect and other between-participant confounding factors, we now used the reinstatement of the non-associated latent task (e.g., E in AC complex task) as a baseline and subtracted it from the reinstatement of the associated latent task (e.g., B in AC complex task). The new analysis revealed a narrower time window that showed reinstatement of the associated latent task than previously reported (850 - 1000ms, as compared to 850 – 2000ms in previous results), allowing us to use the whole time window for the correlation analysis.

7. Please specify that the RTs are residualized in Figure 1.

Response: Following the reviewer's suggestion, we changed our main manuscript, clarifying that the RTs in Figure 1 is residual RTs, not raw RTs. We additionally included raw RTs statistics in the supplemental materials.

Academic Editor:

1. Conceptual framing: The authors refer to ʻtask-basedʼ representations. It seems more appropriate to use the term ʻfeature-basedʼ attention. Basically, the cues, both in simple and in complex tasks indicate the features that are relevant for a particular task. The decoders trained on the simple tasks should thus reflect the features that are attended.

Response: We thank the editor for raising an important issue regarding the term 'task', and its relation to attentional set. We agree with the editor's point that the simple task is strongly intertwined with the stimulus feature participants need to focus on for successful response.

We would like to first clarify that the way we defined tasks is similar to the definition of tasks used by many other influential research on task switching (e.g., Rogers and Monsell, 1995; Rubin and Meiran, 2005; Rubinstein, Meyer, and Evans, 2001). Additionally, given the available evidence regarding mixing costs and the effects of valency of the stimuli, it appears that the switch costs observed in tasks defined on relevant stimulus features do not merely reflect attentional processes, although they likely play a role.

In the revised manuscript, we explicitly defined the term task and discussed its relation with similar terms on p. 5:

While the 'task' in this paradigm is strongly intertwined with feature-based attention, our use of the term is consistent with classic task-switching literature(28-30), where a 'task' is requires the process that enables the efficient guidance of attention toward task-relevant feature. Furthermore, the task-switching literature provides convincing evidence that a 'task' cannot be fully explained by feature-based attention(29-32). For instance, mixing costs, which refer to the cognitive cost observed when performing a task in a mixed-task block (i.e., on each trial one of multiple possible tasks is performed) compared to a single-task block, cannot be fully explained by shift of feature-based attention, since the cost occurs even when participants repeat the same task, implying no change in feature-based attention (29, 30).

2. Experimental design: The design is very elegant. However, as the authors hint at, the features differ in their discernability/difficulty. This is problem for interpreting the decoding results. Moreover, the authors do not state that the different features were equally often the task-relevant features in the generalizable and non-generalizable complex tasks. This is essential to interpret the results. Likewise, they do not state that each feature value was equally frequent.

Response: We thank the editor for the critical feedback regarding the design of the experiment. First ,regarding the features differ in their discernability/difficulty, we performed additional analysis, showing temporal dynamics of decoding probabilities

in decoding analysis and temporal generalization analysis, which will be described more detail in the editor's next comment.

Second, we would like to note that each feature was randomly assigned to each task label (e.g., A,B,C,D,E, and F) across participants. This means that the different features are equally often being the task-relevant features in the generalizable and non-generalizable complex tasks. For further assurance, we conducted an additional analysis, analyzing the number of each simple task assigned to each task label across participants. The result is shown in the figure below. We observed no systematic bias of one feature assigned to specific label highly frequent. This result further suggests that any behavioral or neural effect was not driven by specific feature or simple task.

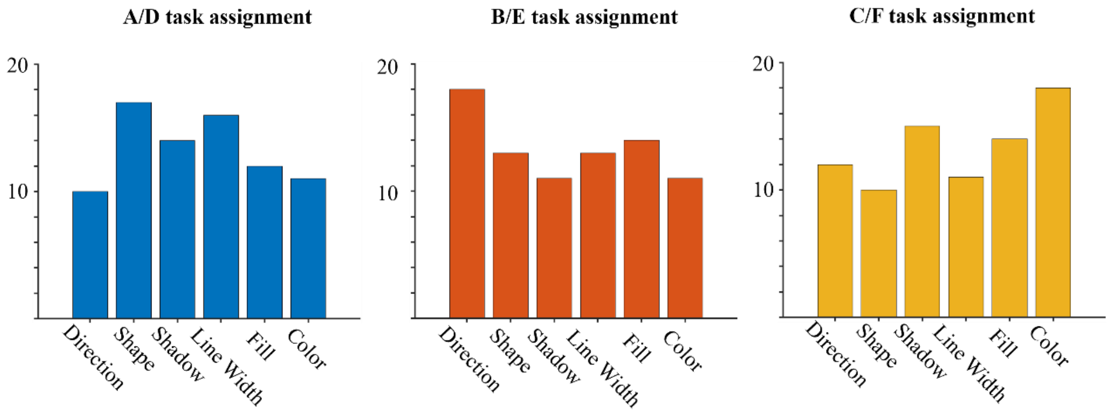

Lastly, we note that all feature value was presented equally frequent across blocks. All information is now included in the supplemental material, Fig S5, and also in the main manuscript, p. 7:

We note that the assignment of each simple task to a specific label (e.g., A, B, C, D, E, or F) was always random for each participant. For additional confirmation, we visualized frequencies of each simple task assigned to each specific label (Fig. S6). This histogram showed no systematic bias of simple task assignments. This result supports that the reported findings are not mainly driven by a particular simple task.

3. The authors show only very derived decoding results. To assess the decoding results, it is essential to present the main basic results.
- Please show the decoding probabilities individually for each simple task (i.e. 6 time courses). If the decoding traces differ in their temporal evolution, this would make interpretation more challenging.
- Could you also please show the decoding probability timecourses (and temporal generalization matrices) for all 6 features.
- The key statistical comparison should then be performed between B (shared component)

and E (not implicated) on trials that include A and C, if previously learnt complex tasks include AB, BC, DE and EF. It is important to directly compare B and E, because only those two components are matched in terms of presentation frequency. Moreover, for those analyses it is essential that the features are rotated across feature-based attention conditions to ensure that potential differences in decoding probability are not driven by stimulus differences (e.g. very often some features are associated with overall higher decoding probability than other features.

Response: We thank the editor for their meticulous comments regarding the decoding results of each simple task and their differences in time courses.

Following the editor's suggestion, we added a decoding probability of each simple task separately with 6 lines along the time points. Additionally, we performed a temporal generalization analysis for each simple task, which provide the detailed information about the dynamics of each simple task representation. These results are reported in the supplemental material in Fig S4 and S5, as you can see below in this section. From these results (Fig. S4 & S5), while we found out that there is a difference of decoding probabilities across simple tasks, there is no systematic bias of decoding probabilities based on the behavioral performance of each task, such that simple tasks with worst performance showing highest, or lowest, decoding performance. Furthermore, temporal dynamics of decoding probabilities (Fig. S5) were highly similar across simple tasks, showing peak at 200-300 ms, matching the result of our cross-phase decoding analysis. Temporal generalization analysis showed the identical pattern, with no observable differences in their temporal dynamics. All showing dynamic coding of simple task representation, peaking at 200-300 ms in both training and testing EEG data.

Secondly, we strongly agree to the editor's comment that to control any frequency effect of latent task or any other potential between-participant confounding factors, it is important to assess latent task activation that is not implicated (E in AC) and use it as a baseline for EEG generalization effect. Following your advice, we directly compared the decoding strength of the associated latent task (e.g., B during AC) against that of the non-associated task (e.g., E during AC) using paired t-test. A cluster-based permutation test revealed that the activation of the relevant latent task B was indeed significantly stronger than that of the non-associated task E during the 850-1,000ms (Fig. 2C; $p_{corrected}$ = .048). We believe this result reflects the EEG generalization effect specific to the associated latent task and not the non-related latent task, while the previous EEG generalization effect (only B in AC) might be confounded by higher presentation frequency of the latent task during the training

stage. Thus, we now use this contrast of associated vs. non-associated latent task as our main EEG test for the generalization effect, and also its results for any additional analyses, including correlational analysis.

Lastly, we agree with the editor's comment that it is important to confirm whether each simple task was evenly distributed across task labels to ensure that the EEG generalization effect is not simply driven by particular simple task(s) being more frequently assigned to a specific task. While we fully randomized the assignment of specific simple task to each task label, it would be critical to directly test if each task is well distributed to task labels across participants. To test this, we performed a confirmatory analysis that shows frequency of each task assigned to specific task label (A,B,C,D,E, or F), and visualized it using histogram (Fig. S6). The results and our interpretation can be found in the response to the previous comment.

We are grateful for these suggestions, which have led to a further strengthening our manuscript.

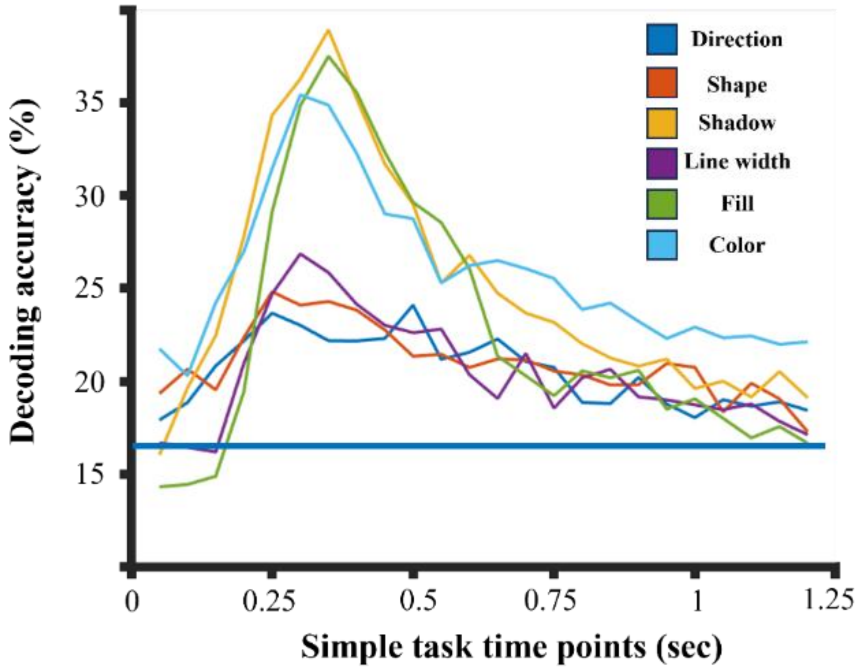

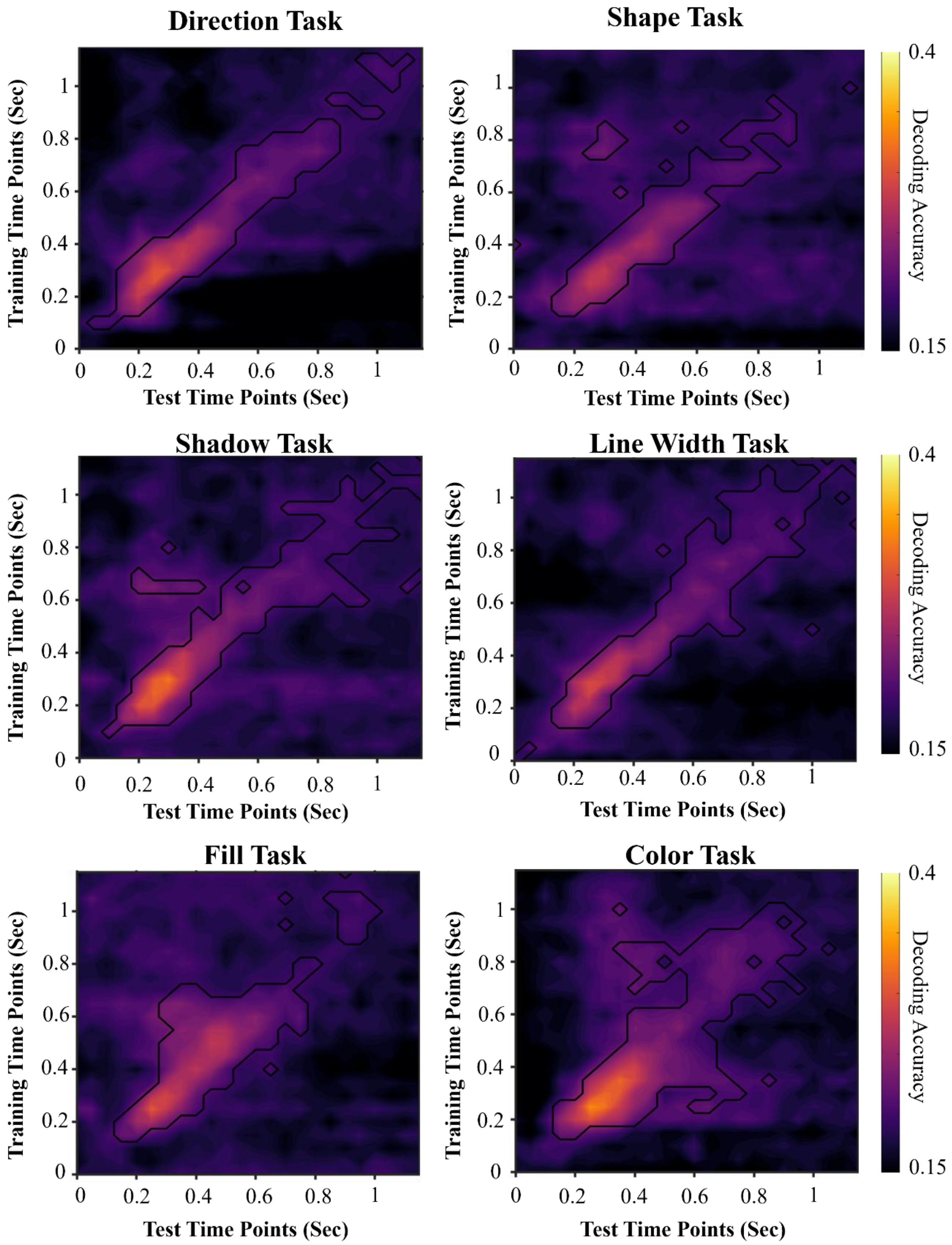

Direction Task
Shape Task
Shadow Task
Line Width Task
Fill Task
Color Task
Training Time Points (Sec)
Test Time Points (Sec)
Decoding Accuracy
0.4
0.15
0
0.2
0.4
0.6
0.8
1
