# Supplementary material for: Neural traces of composite tasks in complex task representation in the human brain reflects learning performance
Source: PLoS Biol. 2026 Jan 16;24(1):e3003613. doi: 10.1371/journal.pbio.3003613 (PMC12826513; doi:10.1371/journal.pbio.3003613)
Supplement: Attachment — Submitted filename: Response_R2.docx [file pbio.3003613.s012.docx]

**Response to editorial and policy-related requests**

We have addressed all the editorial and policy-related requests as follows:

(1) We have changed title from “Decoding task representations that support generalization in hierarchical task” to “Neural traces of composite tasks in complex task representation in the human brain reflects learning performance”.

(2) We have added the ‘Ethics statement’ that includes Institutional Review Board (IRB) approval number, compliance with the Declaration of Helsinki, and acquisition of participants written consent.

(3) We have provided the individual numerical values that underlie the summary data displayed in all figures and custom codes in the archived Open Science Framework (OSF) with the following DOI: [https://doi.org/10.17605/OSF.IO/MZF4A](https://doi.org/10.17605/OSF.IO/MZF4A%20) .

(4) Methods and references previously in the Supplementary Information have been moved to the main manuscript.

(5) Full list of legends for the Supporting Information files has been added to the main manuscript after the references list.

(6) We have added the ‘Financial disclosure statement’, which includes the role of the funder in the study (none).

**Response to Reviewers**

Reviewer #1: The authors extensively revised their paper in response to the points I raised, and carefully considered all of my suggestions. They included multiple additional analyses and revised their framing in light of the new results. In my view, this has substantially strengthened this work. I am happy to recommend publication.

**Response: We appreciate the reviewer’s positive evaluation and recommendation for publication.**

Reviewer #2: The authors have provided excellent responses to my comments. I am happy to recommend the publication of this manuscript!

**Response: We appreciate the reviewer’s positive evaluation and recommendation for publication.**
